# Association of advanced therapies for intermediate- to high-risk pulmonary embolism with improved right ventricular function on outpatient follow-up among survivors

**DOI:** 10.3389/fcvm.2026.1767184

**Published:** 2026-06-10

**Authors:** Hammad Sheikh, Avinash Singh, Connor Smith, Howard Freeman, Abdul Rehman, Jeeyune Bahk, Sakul, Keshav Dixit, Alvin Yang, Maria Riasat, Robert Lookstein, Edgar Argulian, David J. Steiger

**Affiliations:** 1 Division of Pulmonary, Critical Care and Sleep Medicine, Department of Medicine, Icahn School of Medicine at Mount Sinai Health System, New York City, NY, United States; 2 Department of Medicine, TidalHealth Peninsula Regional, Salisbury, MD, United States; 3 Division of Cardiology, Department of Medicine, Icahn School of Medicine at Mount Sinai Health System, New York City, NY, United States; 4 Department of Radiology, Icahn School of Medicine at Mount Sinai Health System, New York City, NY, United States

**Keywords:** echocardiography, embolectomy, pulmonary embolism, right ventricular function (RV function), thrombectomy

## Abstract

**Background:**

The impact of catheter-directed thrombolysis (CDT), catheter-directed embolectomy (CDE) and surgical pulmonary embolectomy (SPE) on long term RV function among patients with acute PE remains unclear. We investigated the association of receipt of advanced therapies with echocardiographic parameters of RV function at outpatient follow-up among survivors of acute PE.

**Methods:**

A retrospective cross-sectional study was performed on all patients with acute PE, who were managed in the Mount Sinai Health System (New York, NY) by PERT between January, 2021 and December, 2023, and had baseline & follow-up echocardiography performed. Non-parametric propensity score matching was performed in R using the *MatchIt* package. A propensity score was calculated from six variables: age, BMI, PESI, ESC risk class, Composite PE Shock score and need for systemic thrombolysis. Multivariable regression models were fitted within the propensity score matched sample incorporating matching variables as interaction factors for a doubly robust estimate. Marginal means and marginal odds ratios were computed using “ATE” (average treatment effect) as the target estimand along with calculation of cluster-robust standard errors. *P*-values were adjusted for multiple comparisons using the Benjamini-Hochberg procedure. An adjusted *p*-value cut-off of 0.05 was chosen for statistical significance.

**Results:**

A total of 122 patients (70 male) were included with median age 64.4 years, median PESI score 86 points and ESC risk group intermediate-low (26 [21.3%]), intermediate-high (76 [62.3%]) and high (20 [16.4%]) risk respectively. CDE, CDT and SPE were performed in 21 (17.2%), 11 (9.0%) and 11 (9.0%) cases. In matched analyses, receipt of any advanced therapy (compared to anticoagulation alone) was associated with increased RVOT VTI (4.6 cm; *p* = 0.009) and RV Ś (2.0 cm/s; *p* = 0.006) as well as higher odds of TR grade improvement (marginal OR: 3.0; *p* = 0.002).

**Conclusion:**

Receipt of advanced therapies for acute PE was associated with statistically significant improvement in echocardiographic parameters of RV function at outpatient follow-up (median 26 weeks) among survivors of acute PE compared to systemic anticoagulation alone. Given the retrospective, non-randomized design, modest sample size, and use of surrogate endpoints, these findings should be considered hypothesis-generating and warrant confirmation in prospective studies incorporating clinical, functional, and patient-centered outcomes.

## Introduction

Pulmonary embolism (PE) is the third most common acute vascular disease worldwide after myocardial infarction and cerebrovascular accident (1). In the United States, hospitalizations for acute PE have steadily increased over the prior two decades along with associated increases in hospital charges (2). Mortality from acute PE varies widely depending on the severity of PE (3). Based on guidelines from the European Society of Cardiology (ESC), high-risk PE is defined as hemodynamically unstable PE, while hemodynamically stable patients with biochemical, radiographic and/or echocardiographic evidence of right heart strain are considered intermediate-risk (4). The overall in-hospital all-cause mortality rate of acute PE in the United States is approximately 6.0%, which can reach up to 42% for patients with high-risk PE (3). Additionally, among survivors of acute PE, post-PE syndrome is a recognized clinical entity characterized by functional impairments in gas exchange, decreased quality of life and significant morbidity (5). Recent evidence suggests that even among children and young adults diagnosed with acute PE, one-third of patients had a significant decline in exercise capacity and impairments on cardiopulmonary exercise testing at long-term (1–2 years) follow-up (6). As such, the long-term impact of acute PE is being increasingly recognized and the need to address this aspect of PE care cannot be overemphasized. Real-world registry data further underscore the healthcare burden of acute PE. In the RIETE registry analysis (7), hospitalization practices and duration of hospitalization for acute PE varied substantially across countries, highlighting both the frequency with which PE requires inpatient care and the heterogeneity of real-world resource utilization. These data reinforce the clinical importance of identifying therapeutic strategies that may improve early stabilization, shorten downstream morbidity, and optimize post-discharge recovery among appropriately selected patients.

The optimal treatment of intermediate-risk and high-risk acute PE remains incompletely defined. Historically, systemic thrombolysis (ST) has been considered the standard of care for high-risk PE, while systemic anticoagulation alone has been recommended for patients with low-risk PE (4). Surgical pulmonary embolectomy (SPE) has been traditionally reserved for patients with high-risk PE who have either failed ST or have contraindications to it (8). However, recent studies have shown that SPE may afford superior pulmonary outcomes in unselected patients with high-risk PE as compared to those receiving ST alone (9). Moreover, in the PIETHO trial, ST for patients with intermediate-risk acute PE was shown to reduce death or hemodynamic compensation at 7 days (compared to placebo) but with an increased risk of hemorrhagic stroke or major bleeding complications (10). Percutaneous catheter-directed thrombolysis (CDT) and catheter-directed embolectomy (CDE) were developed with the aim to improve hemodynamic outcomes in patients with intermediate- or high-risk PE while affording a reduced risk of major bleeding (11). CDE is considered to be a less invasive alternative to SPE for patients with intermediate & high-risk PE and has been shown to have comparable 30-day mortality and major bleeding rates (12). Additionally, interim results of the FLASH registry showed that CDE performed for unselected patients with intermediate- or high-risk PE was associated with a low (0.4%) 30-day all-cause mortality rate (13). Moreover, results of the recent STORM-PE trial demonstrated that CDE for intermediate high-risk PE resulted in greater acute improvements in RV/LV ratio without a concomitant increase in 7-day major adverse events (14). More recent randomized and registry data have begun to refine the role of catheter-based strategies in selected patients with intermediate-risk PE. In HI-PEITHO, a multinational randomized trial of 544 patients with acute intermediate-risk PE and additional indicators of cardiorespiratory distress, ultrasound-facilitated catheter-directed fibrinolysis plus anticoagulation reduced the 7-day composite of PE-related death, cardiorespiratory decompensation or collapse, or symptomatic recurrent PE compared with anticoagulation alone (15). Major bleeding was numerically higher in the intervention group but not statistically different at 7 or 30 days. In parallel, PRAGUE-26 is evaluating simple catheter-directed thrombolysis vs. anticoagulation alone in intermediate-high-risk PE, with clinical, functional, patient-reported, and cost-effectiveness outcomes over longer follow-up (16). Despite these advances, data remain limited regarding whether advanced reperfusion therapies are associated with recovery of RV function beyond the acute hospitalization, particularly when assessed using standardized follow-up echocardiography (11).

We hypothesized that patients with intermediate- to high-risk acute PE who underwent specific advanced reperfusion therapies (CDE, CDT or SPE) may have greater improvements in RV function at outpatient follow-up—as assessed by echocardiographic parameters—when compared to patients who did not receive any advanced reperfusion therapy (ART). In the present study, our aim was to study the association of improvement in echocardiographic parameters of RV function at outpatient follow-up among patients with intermediate- to high-risk PE, who received any ART vs. those patients who did not receive any ART.

## Patients and methods

A retrospective cross-sectional study was performed after obtaining approval from the IRB at Icahn School of Medicine at Mount Sinai (New York City, NY) with approval number IRB STUDY-18-00725. Given that no direct patient contact was involved for this study, the requirement of informed consent was waived by the institutional review board for this retrospective chart review in line with 45 CFR §46.104(d)(4). Medical records were accessed by study personnel between 1st December, 2024 and 31st December, 2024. Data recorded on standardized spreadsheet by study personnel did not contain any patient identifying information and was password-protected.

All patients, who were diagnosed with intermediate- or high-risk acute PE from January, 2021 to December, 2023 at one of three urban teaching hospitals (viz. Mount Sinai Morningside, Mount Sinai West and Mount Sinai Beth Israel) in the Mount Sinai Health System, were eligible for inclusion in the study. We excluded patients who: (a) did not undergo echocardiography within 24 h of the diagnosis of acute PE; (b) did not have follow-up echocardiography at least 2 months after discharge from the hospital; or (c) were under eighteen years of age.

### Diagnosis of PE and risk stratification

In all patients, acute PE was diagnosed by visualization of filling defects within the pulmonary arteries on pulmonary computed tomographic angiography (CTA). For risk stratification of acute PE, guidelines from the ESC were utilized. High-risk PE was defined as acute PE with: (i) sustained hypotension for 20 min despite fluid resuscitation; or (ii) shock requiring the use of vasopressors to maintain a systolic blood pressure of 90 mm Hg. Intermediate high-risk PE was defined as acute PE with the presence of both biochemical and imaging (echocardiographic or radiographic) evidence of right heart strain in the absence of hemodynamic instability. Intermediate low-risk PE was defined as hemodynamically stable acute PE with either positive biomarkers (elevated troponin I or brain natriuretic peptide) or imaging (echocardiographic or radiographic) evidence of right heart strain—but not both—in a hemodynamically stable patient. In line with recently published literature, we also calculated the Composite PE Shock (CPES) score given its ability to identify PE patients in normotensive shock (17, 18).

### PERT-based care

Within our network of hospitals, all patients diagnosed with intermediate- or high-risk acute PE were managed by a dedicated PE Response Team (PERT) (19). In brief, the PERT consisted of the on-call pulmonologist, cardiologist, intensivist, interventional radiologist and cardiothoracic surgeon. A PERT consult was initiated by the patient's primary attending physician (usually, ED attending or hospitalist) and the patient's plan of care was formulated after mutual discussion between members of the PERT and the patient's primary attending. Although PERT was mostly consulted for intermediate- or high-risk acute PE, a PERT consult could be requested for patients with intermediate low-risk acute PE at the discretion of the primary attending (usually, in the presence of severe co-existent illness or significant medical comorbidities). Systemic anticoagulation was administered to all patients in the absence of contraindications (20). Inferior vena cava (IVC) filter insertion and extracorporeal membrane oxygenation were also offered to selected patients.

### Advanced reperfusion therapies for PE

Within the Mount Sinai Health System, ART offered to patients included CDE, CDT and SPE.

CDT and CDE were performed by two interventional radiologists with more than twenty years of experience in the field of interventional radiology. CDT was performed by establishing percutaneous central venous access and placing bilateral pulmonary artery catheters. Alteplase bolus was administered through the catheters followed by a slow infusion of alteplase over 16‒24 h. Fibrinogen levels were used to titrate the rate of alteplase infusion. After completion of CDT, pulmonary angiography was performed to assess if CDT was successful. CDE was performed using the FlowTriever® (Inari Medical, Irvine, CA)—an over-the-wire mechanical thrombectomy device—after establishing percutaneous central venous access. In selected patients, both CDT and CDE were administered at the discretion of PERT.

SPE was performed by a single cardiothoracic surgeon with more than twenty years of experience in the field of cardiothoracic surgery. Indications for SPE were failed CDT or CDE, significant hemodynamic instability precluding percutaneous interventions, paradoxical embolism, clot-in-transit or probable signs of chronic thromboembolic pulmonary hypertension necessitating surgical intervention. SPE was performed by a midline sternotomy approach followed by bi-caval cannulation and total cardiopulmonary bypass. Direct extraction of clots from the pulmonary trunk and main pulmonary arteries was performed. In selected patients, the incision was extended and/or additional incisions were made to extract intracardiac thrombi (clot-in-transit).

### Echocardiography and echocardiographic parameters

Formal transthoracic echocardiography was performed in all patients by either cardiology fellows or certified echocardiography technicians using standard transthoracic echocardiography platforms (Philips CX50; Philips Medical Systems, Andover, MA; GE Vivid S70, GE Healthcare, Milwaukee, WI). The minimum dataset for interpretation included the standard parasternal long-axis (PLAX), parasternal short-axis (PSAX) and apical RV focused (RVF) views. For this study, each echocardiography scan was re-interpreted in a structured manner by a single cardiology fellow, who was blinded to the clinical history and outcome of patients as well as the original echocardiography interpretation. For this purpose, DigiView® (Intelerad Medical Systems, Inc., Raleigh, NC) software was used on standard diagnostic workstations. Guidelines from the American Society of Echocardiography for the assessment of right heart in adults were considered as the reference standard (21). Basal and mid-RV diameters were recorded at end-diastole in RVF view. RV outflow tract (RVOT) diameter was recorded at end-diastole in PSAX view. Right atrial (RA) area was recorded at end-systole in RVF view (Simpson's method of disks), while RV fractional area change (FAC) was calculated from measurements of the RV area at end-systole and end-diastole in RVF view. RVOT acceleration time (RVOT AT) and RVOT velocity time integral (VTI) or stroke distance on pulsed-wave Doppler were measured on PSAX view by placing the sampling volume just below the pulmonic valve on the RV side. Tricuspid regurgitant (TR) jet maximum velocity (V_max_) was measured using continuous wave Doppler in multiple views and the highest velocity was recorded. Simplified Bernoulli equation was used to calculate the TR pressure gradient from TR jet V_max_, which was combined with the IVC-based estimated RA pressure to obtain a measurement of pulmonary artery systolic pressure (PASP). Severity of TR was also graded qualitatively as “none”, “mild”, “moderate” or “severe”. Tricuspid annular plane systolic excursion (TAPSE) was recorded on M-mode echocardiography in RVF view, while RV Ś (lateral tricuspid annulus peak velocity) was recorded on tissue Doppler imaging in RVF view. Presence of McConnell sign (RV apical hyperkinesia with free wall akinesia) on RVF view and left-sided valvular disease were also recorded. In the case of technically difficult windows or poor-quality images precluding accurate measurements, missing values were recorded for those echocardiographic parameters.

### Statistical analysis

Statistical analysis was performed in R version 4.1.1. Median and interquartile ranges (IQR) were calculated for quantitative variables and frequencies were computed for qualitative variables. A propensity score-matched analysis was performed using the *MatchIt* package for R with the optimal *full* matching specification (22). This package implements the optimal *full* matching as a form of non-parametric matching using the propensity score to first stratify patients into clusters and then, computing a stratum propensity score to estimate weights for each patient in the cluster. The propensity score was calculated from six variables (*viz*. age, body mass index [BMI], PESI [PE Severity Index] score, ESC risk class, CPES score and ST) using a generalized linear model with a *probit* link function. Matching was performed on the propensity score using the optimal *full* matching specification with the target estimand being average treatment effect in the entire population (“ATE”). To ensure adequate balance in the matched sample, both quantitative (including standardized mean differences on matched variables) and qualitative measures (Love plots and density plots) were assessed. Mean differences in quantitative echocardiographic parameters were calculated using regression estimation (also known as *g*-computation), which incorporated the grouping variable and other covariates as interaction terms in the regression model to provide doubly robust estimates. Cluster-robust standard errors were computed for regression models using the *marginaleffects* package for R. For all comparisons, *p*-values were adjusted for multiple comparisons using the modified Bonferroni correction method described by Hochberg (23). An adjusted *p*-value cut-off of 0.05 was chosen for statistical significance.

## Results

**Figure 1:**
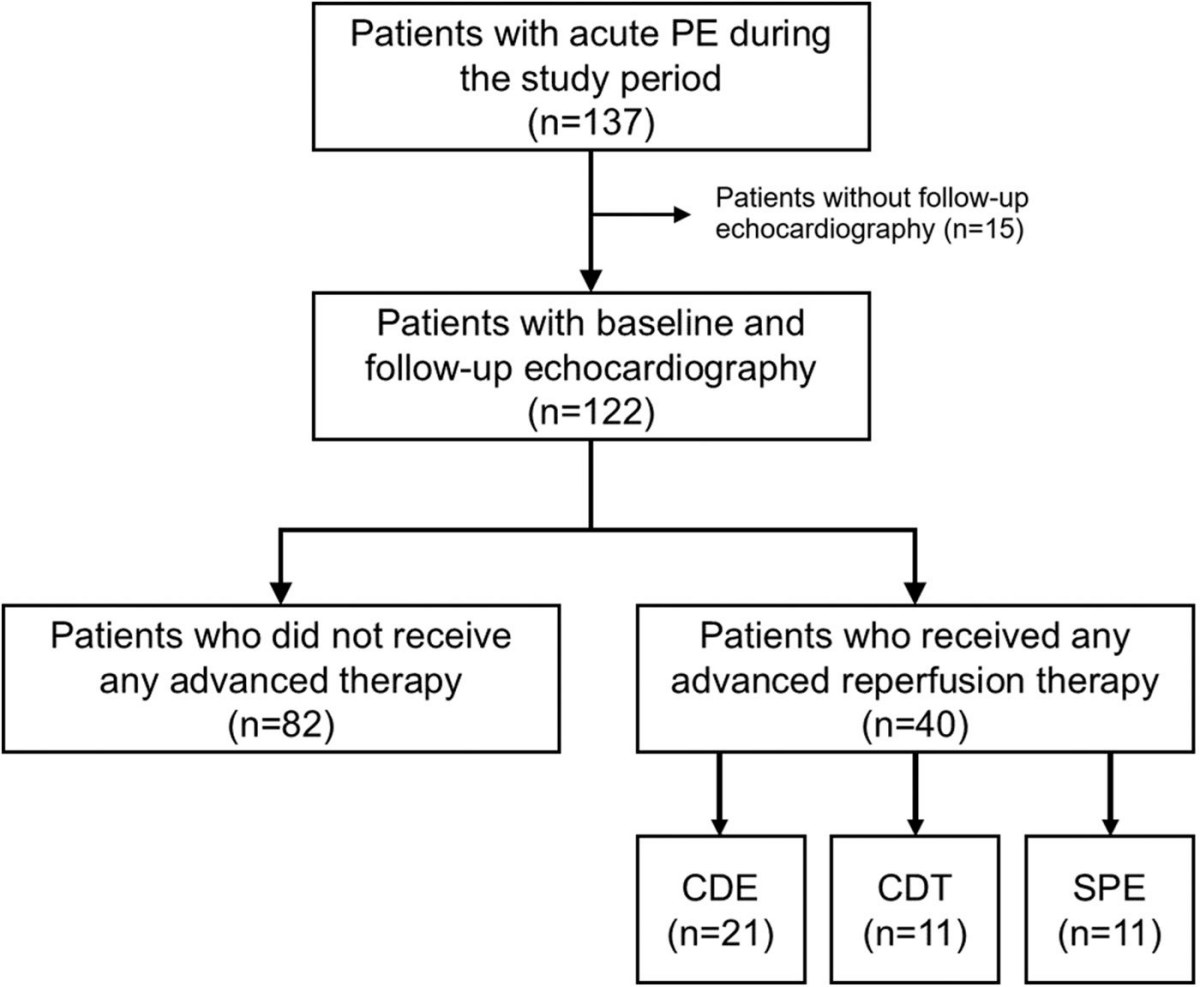
A flowchart depicting the inclusion and exclusion of patients. *CDE*, Catheter-directed embolectomy; *CDT*, catheter-directed thrombolysis; *SPE*, surgical pulmonary embolectomy.

A total of 122 patients met the inclusion criteria and were included in the final analysis (details in Figure 1). A slight predominance of male patients (*n* = 70, 57.4%) was observed in the sample. The median age of included participants was 64.4 (IQR: 52.5‒74.7) years and 52 (42.6%) were female. White (*n* = 62, 50.8%) and Black (*n* = 56, 45.9%) patients were the main racial groups described in the study. Only a small proportion of patients (*n* = 8, 6.6%) were of Hispanic or Latino ethnicity and only 5 (4.1%) patients designated Spanish as their preferred language. Most patients (*n* = 116, 95.1%) had some form of medical insurance with private insurance (*n* = 36, 29.5%), commercial Medicare (*n* = 32, 26.2%) and government Medicare (*n* = 24, 19.7%) being the most common payors. The median BMI of included patients was 29.0 (IQR: 25.2‒35.4) kg/m^2^ and the median PESI score was 86 (IQR: 69‒99) points. With respect to ESC risk group, 26 (21.3%), 76 (62.3%) and 20 (16.4%) patients were intermediate low-risk, intermediate high-risk and high-risk respectively. The median CPES score for our patients was 3 (IQR: 3‒4). Further details are provided in Table 1.

**Table 1:** Demographic characteristics and clinical features of patients included in the study (*n* = 122).

Characteristics	Results
Age (median [IQR]), years	64.4 (52.5‒74.7)
Sex	
Female	52 (42.6%)
Male	70 (57.4%)
Race	
White	62 (50.8%)
Black	56 (45.9%)
Asian	3 (2.5%)
American Indian	1 (0.8%)
Insurance	
Government Medicare	24 (19.7%)
Government Medicaid	10 (8.2%)
Commercial Medicare	32 (26.2%)
Commercial Medicaid	14 (11.5%)
Private insurance	36 (29.5%)
None	6 (4.9%)
Ethnicity	
Hispanic or Latino	8 (6.6%)
Non-Hispanic/Latino	114 (93.4%)
Preferred language	
English	117 (95.9%)
Spanish	5 (4.1%)
Body mass index, kg/m^2^	29.0 (25.2‒35.4)
Past medical history	
Prior episode of VTE	27 (22.1%)
On anticoagulation	4 (3.3%)
Cardiac disease	5 (4.1%)
Asthma or COPD	16 (13.1%)
Pulmonary hypertension	2 (1.6%)
Risk factors for PE	
Active malignancy	14 (11.5%)
Immobility for ≥3 days	9 (7.4%)
Surgery within 4 weeks	6 (4.9%)
Predisposing medications	7 (5.7%)
Recent long flight or travel	7 (5.7%)
Family history of thrombophilia	7 (5.7%)
Symptoms	
Pleuritic chest pain	52 (42.6%)
Hemoptysis	3 (2.5%)
Dyspnea	98 (80.3%)
Syncope	26 (21.3%)
Unilateral leg swelling	29 (23.8%)
Clinical signs on presentation	
Tachycardia	62 (50.8%)
Low systolic blood pressure	2 (1.6%)
Tachypnea	61 (50.0%)
Hypoxia	10 (8.2%)
PESI score (median [IQR])	86 (69‒99)
CPES score (median [IQR])	3 (3‒4)
ESC risk group	
Intermediate low-risk	26 (21.3%)
Intermediate high-risk	76 (62.3%)
High-risk	20 (16.4%)
Site where PE diagnosed	
Emergency department	115 (94.3%)
Inpatient	7 (5.7%)
Disposition on admission	
Intensive care unit	109 (89.3%)
Step down unit	6 (4.9%)
Telemetry ward	1 (0.8%)
Medical or surgical floor	6 (4.9%)
Location of PE	
Saddle (central)	27 (22.1%)
Main pulmonary artery (central)	60 (49.2%)
Lobar branch (central)	83 (68.0%)
Segmental branch (peripheral)	56 (45.9%)
Subsegmental (peripheral)	44 (36.1%)
Associated CT findings	
Signs of right heart strain	107 (87.7%)
Pulmonary infarct	20 (16.4%)
Pleural effusion	12 (9.8%)
Venous Doppler study performed	91 (75.6%)
Findings of venous Doppler	
CFV thrombosis	13 (10.7%)
SFV thrombosis	7 (5.7%)
Popliteal vein thrombosis	28 (22.9%)
Other distal DVT	1 (0.8%)
Superficial vein thrombosis	2 (1.6%)
Laboratory investigations (median [IQR])	
D-dimer, mg/L	11.2 (5.56‒20.0)
Troponin I, ng/mL	0.141 (0.045‒0.373)
Brain natriuretic peptide, pg/mL	153 (54‒329)
Blood urea nitrogen, mg/dL	17 (12‒23)
Red cell distribution width, %	12.8 (12.0‒14.5)
Serum creatinine, mg/dL	1.02 (0.86‒1.27)

COPD, Chronic obstructive pulmonary disease; CPES, Composite Pulmonary Embolism Shock; CFV, common femoral vein; CT, computed tomography; DVT, deep venous thrombosis; ESC, European Society of Cardiology; IQR, interquartile range; PE, pulmonary embolism; PESI, PE Severity Index; SFV, superficial femoral vein; VTE, venous thromboembolism.

The most common presenting symptoms in our patients were dyspnea (*n* = 98, 80.3%), pleuritic chest pain (*n* = 52, 42.6%), unilateral leg pain or swelling (*n* = 29, 23.8%) and syncope (*n* = 26, 21.3%). Only a small proportion of patients had identifiable risk factors for acute PE, such as active malignancy (*n* = 14, 11.5%) and immobility for three or more days (*n* = 9, 7.4%). With respect to past medical history, 27 (22.1%) patients had a history of prior VTE and 4 (3.3%) patients were on anticoagulation prior to diagnosis of acute PE. A small proportion of patients had a prior history of chronic lung disease (*n* = 16, 13.1%), congestive heart failure (*n* = 5, 4.1%) and pulmonary hypertension (*n* = 2, 1.6%).

Upon presentation, 62 (50.8%), 61 (50.0%), 10 (8.2%) and 2 (1.6%) had tachycardia, tachypnea, hypoxia and hypotension respectively. PE was diagnosed incidentally in 3 (2.5%) patients, while another 4 (3.3%) patients had hospital-acquired PE. Saddle PE was present in 27 (22.1%) cases and central PE was noted in 117 (95.9%) CTA scans. Evidence of right heart strain was noted on 107 (87.7%) CTA scans. Lower extremity venous Doppler studies were performed in 91 (75.6%) cases and evidence of lower extremity deep vein thrombosis (DVT) was present on 51 (41.8%) studies. Median values for troponin I and brain natriuretic peptide were 0.141 (IQR: 0.045‒0.373) ng/ml and 153 (IQR: 54‒329) pg/ml respectively. With respect to treatment, systemic anticoagulation was administered to all patients and included unfractionated heparin (*n* = 99, 81.1%) & enoxaparin (*n* = 23, 18.8%). ST was given to 9 (7.4%) patients, while ART were administered in 40 (32.8%) cases. CDE, CDT and SPE were performed in 21 (17.2%), 11 (9.0%) and 11 (9.0%) cases. At the time of discharge, direct-acting oral anticoagulants were the most commonly prescribed anticoagulant (*n* = 102, 83.6%) followed by enoxaparin (*n* = 9, 7.4%) and warfarin (*n* = 6, 4.9%). The median hospital LOS (length of stay) was 6.0 (IQR: 3.6‒9.6) days. The in-hospital rates of bleeding of any severity and major bleeding were 6.6% (8/122) and 3.3% (4/122) respectively. The rate of follow-up in primary care clinics after discharge was 91.0% (*n* = 111), while the rates of follow-up in pulmonary and hematology clinics were 37.7% (*n* = 46) and 18.8% (*n* = 23) respectively. Further details are provided in Table 2.

**Table 2:** Details of treatment and overall clinical outcomes of patients included in the study (*n* = 122).

Characteristics	Results
Systemic anticoagulation	122 (100%)
Initial anticoagulant	
Unfractionated heparin	99 (81.1%)
Low molecular weight heparin	23 (18.8%)
IVC filter insertion	21 (17.2%)
Systemic thrombolysis	9 (7.4%)
Catheter-directed thrombolysis	11 (9.0%)
Catheter-directed embolectomy	21 (17.2%)
Surgical embolectomy	11 (9.0%)
Anticoagulant prescribed upon discharge	
Apixaban	89 (72.9%)
Dabigatran	2 (1.6%)
Rivaroxaban	11 (9.0%)
Warfarin	6 (4.9%)
Enoxaparin	9 (7.4%)
Clinical outcomes	
In-hospital bleeding of any severity	8 (6.6%)
In-hospital major bleeding	4 (3.3%)
Thirty-day bleeding of any severity	14 (11.5%)
Thirty-day major bleeding	6 (4.9%)
Hospital LOS (median [IQR]), days	6.0 (3.6‒9.6)
Outpatient follow-up	
Primary care	111 (91.0%)
Pulmonary	46 (37.7%)
Hematology	23 (18.8%)

LOS, Length of stay; PE, pulmonary embolism; PERT, PE Response Team; IQR, interquartile range; IVC, inferior vena cava.

### Echocardiographic parameters between advanced therapy and control groups

Transthoracic echocardiography at the time of diagnosis of PE was performed in all patients and the median time interval between the diagnosis of PE and echocardiography was 12 (IQR: 4‒21) hours. Follow-up transthoracic echocardiography was performed after discharge in all patients and the median time interval between the initial (inpatient) echocardiography and the follow-up outpatient study was 26.6 (IQR: 10.8‒49.3) weeks. For exploring the association between echocardiographic parameters and receipt of ART, we first compared baseline variables between the two groups (i.e., ART and control groups). The results of these baseline variables are presented in Table 3, which showed that CPES score was higher in the ART group (*p* = 0.012) and PESI score was higher in the control group (*p* = 0.024). Within the unmatched sample, a higher proportion of patients in the advanced therapy group had improvement in TR grade as compared to control group (57.7% *vs.* 21.9%; *p* < 0.001). Additionally, as shown in Table 4, patients in the advanced therapy group had greater improvement in RVOT VTI i.e., stroke distance (+5.825 *vs.* + 2.188; *p* = 0.001) and RV Ś i.e., lateral tricuspid annulus peak systolic velocity (+2.423 *vs.* −0.125; *p* = 0.010).

**Table 3:** Comparison of key baseline variables and outcomes between the advanced therapy and control groups in the unmatched sample (*n* = 122).

Characteristics	Advanced therapy (*n* = 40)	Control (*n* = 82)	*p*-value*
Age (median [IQR]), years	60 (48.7–67.2)	67.5 (55–76)	**0** **.** **019**
Sex			0.999
Female	17 (42.5%)	35 (42.7%)	
Male	23 (57.5%)	47 (57.3%)	
Body mass index, kg/m^2^	30.6 (26.5–37.5)	28.6 (24.4–33.8)	0.106
PESI (median [IQR])	72 (61–90)	89 (73–103)	**0** **.** **004**
ESC risk group, n (%)			**0.033**
Intermediate low	7 (17.5%)	19 (23.2%)	
Intermediate high	31 (77.5%)	45 (54.9%)	
High^a^	2 (5.0%)	18 (21.9%)	
CPES (median [IQR])	4 (3–5)	3 (2–4)	**0** **.** **001**
Systemic thrombolysis, *n* (%)	4 (10.0%)	5 (6.1%)	0.473
Clinical outcomes^a^			
In-hospital bleeding of any severity	3 (7.5%)	5 (6.1%)	0.716
In-hospital major bleeding	2 (5.0%)	2 (2.4%)	0.999
Thirty-day bleeding of any severity	5 (12.5%)	9 (11.0%)	0.771
Thirty-day major bleeding	2 (5.0%)	4 (4.9%)	0.999
Hospital LOS (median [IQR]), days	6.6 (4.4–8.3)	5.9 (3.1–10.3)	0.692

* *P* values derived from Wilcoxon rank-sum test and Fisher's exact test for comparing quantitative and categorical variables, respectively, between the two groups.

a Our cohort included only survivors of acute pulmonary embolism—patients who were able to undergo follow-up echocardiography as an outpatient; patients who died in-hospital or prior to follow-up echocardiography were not captured in this cohort.

CPES, Composite Pulmonary Embolism Shock; ESC, European Society of Cardiology; IQR, interquartile range; LOS, Length of stay; PE, pulmonary embolism; PESI, PE Severity Index; IQR, interquartile range.

Statistically significant differences are highlighted in bold text.

**Table 4:** Change in echocardiographic parameters of RV function among the advanced therapy and control groups in the unmatched sample (*n* = 122).

Quantitative Parameters		Group	PRE		POST		*Δ*			*P* *
			Median (IQR)		Median (IQR)		Mean			
RV basal diameter (cm)		Intervention	4.60 (4.00‒5.00)		3.60 (3.10‒3.72)		−0.977			0.337
		Control	4.30 (3.85‒4.80)		3.70 (3.30‒4.20)		−0.600			
RV mid-diameter (cm)		Intervention	3.60 (2.80‒4.10)		2.75 (2.37‒3.10)		−0.871			0.369
		Control	3.40 (2.90‒3.90)		2.80 (2.30‒3.20)		−0.677			
RVOT diameter (cm)		Intervention	2.60 (2.20‒3.00)		2.45 (2.20‒2.80)		−0.119			0.763
		Control	2.55 (2.30‒2.97)		2.60 (2.25‒2.80)		−0.071			
RVOT PW AT (msec)		Intervention	67.5 (53.2‒85.0)		100 (85.0‒118)		+25.46			0.337
		Control	72.0 (51.0‒90.0)		85.0 (60.0‒109)		+14.95			
RVOT VTI (cm)		Intervention	9.5 (8.2‒10.8)		15.7 (15.1‒16.2)		+5.852			**0.001**
		Control	9.9 (9.1‒10.9)		12.0 (10.6‒13.4)		+2.188			
TR jet V_max_ (m/s)		Intervention	2.96 (2.46‒3.20)		2.30 (1.90‒2.35)		−0.684			0.207
		Control	3.00 (2.50‒3.47)		2.60 (2.30‒3.05)		−0.248			
RAP (mm Hg)		Intervention	5 (4‒8)		5 (4‒5)		−1.887			0.540
		Control	5 (5‒5)		5 (5‒5)		−1;403			
PASP (mm Hg)		Intervention	40.0 (29.3‒51.0)		26.2 (21.4‒29.1)		−13.8			0.317
		Control	42.7 (32.6‒55.7)		36.4 (28.0‒48.6)		−7.00			
TAPSE (cm)		Intervention	1.64 (1.48‒2.00)		2.20 (1.62‒2.47)		+0.274			0.207
		Control	1.87 (1.37‒2.10)		1.98 (1.79‒2.40)		+0.092			
RV Ś (cm/s)		Intervention	9.78 (8.00‒11.5)		12.0 (10.0‒13.3)		+2.423			**0.010**
		Control	12.0 (9.0‒14.2)		11.8 (10.0‒14.0)		−0.125			
RV diastolic area (cm^2^)		Intervention	23.6 (21.0‒29.3)		18.1 (16.6‒21.7)		−5.575			0.337
		Control	23.7 (20.1‒28.9)		20.2 (18.3‒24.0)		−3.797			
RV FAC (%)		Intervention	28.7 (25.9‒34.9)		40.0 (33.0‒44.7)		+8.322			0.658
		Control	29.8 (21.1‒37.3)		37.0 (30.0‒44.0)		+6.648			
RA Area (cm^2^)		Intervention	18.3 (14.0‒21.8)		17.2 (15.0‒19.1)		−1.000			0.369
		Control	17.0 (14.1‒21.2)		15.5 (12.0‒17.8)		−3.000			
**QUALITATIVE PARAMETERS**		**GROUP**	**PRE**		**POST**		**BETTER**			***P*** *
			**N**	**%**	**N**	**%**	**N**	**%**		
McConnell sign		Intervention	25	62.5%	2	5.0%	23	57.5%		0.302
		Control	39	47.6%	6	7.3%	33	40.2%		
TR grade	None	Intervention	6	15.0%	22	55.0%	I	23	57.5%	**<0.001**
		Control	13	15.8%	29	35.4%				
	Mild	Intervention	27	67.5%	18	45.0%				
		Control	46	56.1%	30	36.6%				
	Moderate	Intervention	7	17.5%	0	0.0%	C	18	21.9%	
		Control	14	17.1%	16	19.5%				
	Severe	Intervention	0	0.0%	0	0.0%				
		Control	9	11.0%	7	8.5%				

* Benjamini-Hochberg adjusted *p*-values derived from Wilcoxon rank-sum test and Fisher's exact test for comparing quantitative (*Δ* echocardiographic parameters) and categorical variables (improvement in echocardiographic improvements), respectively, between the two groups.

AT, Acceleration time; C, control group; FAC, fractional area change; I, intervention group; IQR, interquartile range; PASP, pulmonary artery systolic pressure; PW, pulse-wave Doppler; RA, right atrium; RAP, RA pressure; RV, right ventricle; RVOT, right ventricular outflow tract; TAPSE, tricuspid annular plane systolic excursion; TR, tricuspid regurgitation; V_max_, maximum velocity.

Statistically significant differences are highlighted in bold text.

**Figure 2:**
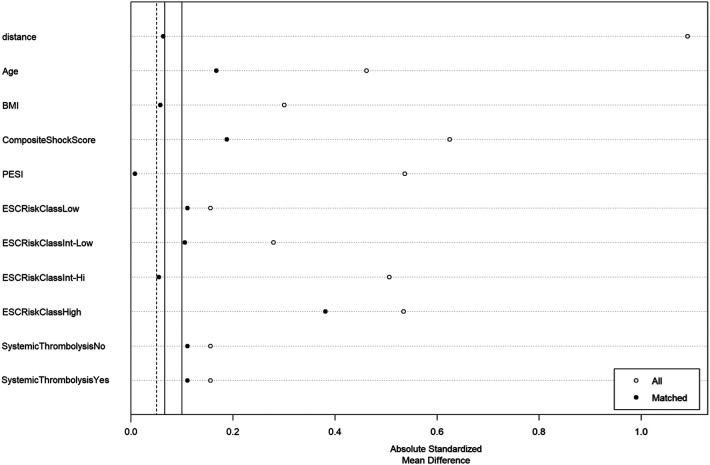
Love plot of absolute standardized mean differences on matching variables between the target and control groups. Matching variables included age, BMI, PESI score, ESC risk group, Composite PE Shock score and systemic thrombolysis. Target group consisted of patients who received CDE, CDT or SPE, while the control group consisted of patients who did not receive any advanced therapy. *BMI*, Body mass index; *ESC*, European Society of Cardiology; *PE*, pulmonary embolism; *PESI*, PE Severity Index.

Given imbalance in baseline variables between the two groups, we performed propensity score-matched analyses. Patients who had received ART were designated as the target group and patients in the control group were matched on the propensity score to one or more patients in the target group using the optimal *full* matching specification—with the desired estimand being the average treatment effect (“ATE”) in the population. The effective sample size of the propensity score-weighted sample was 73.0. Love plot showed adequate balance in the matched sample (Figure 2).

**Figure 3:**
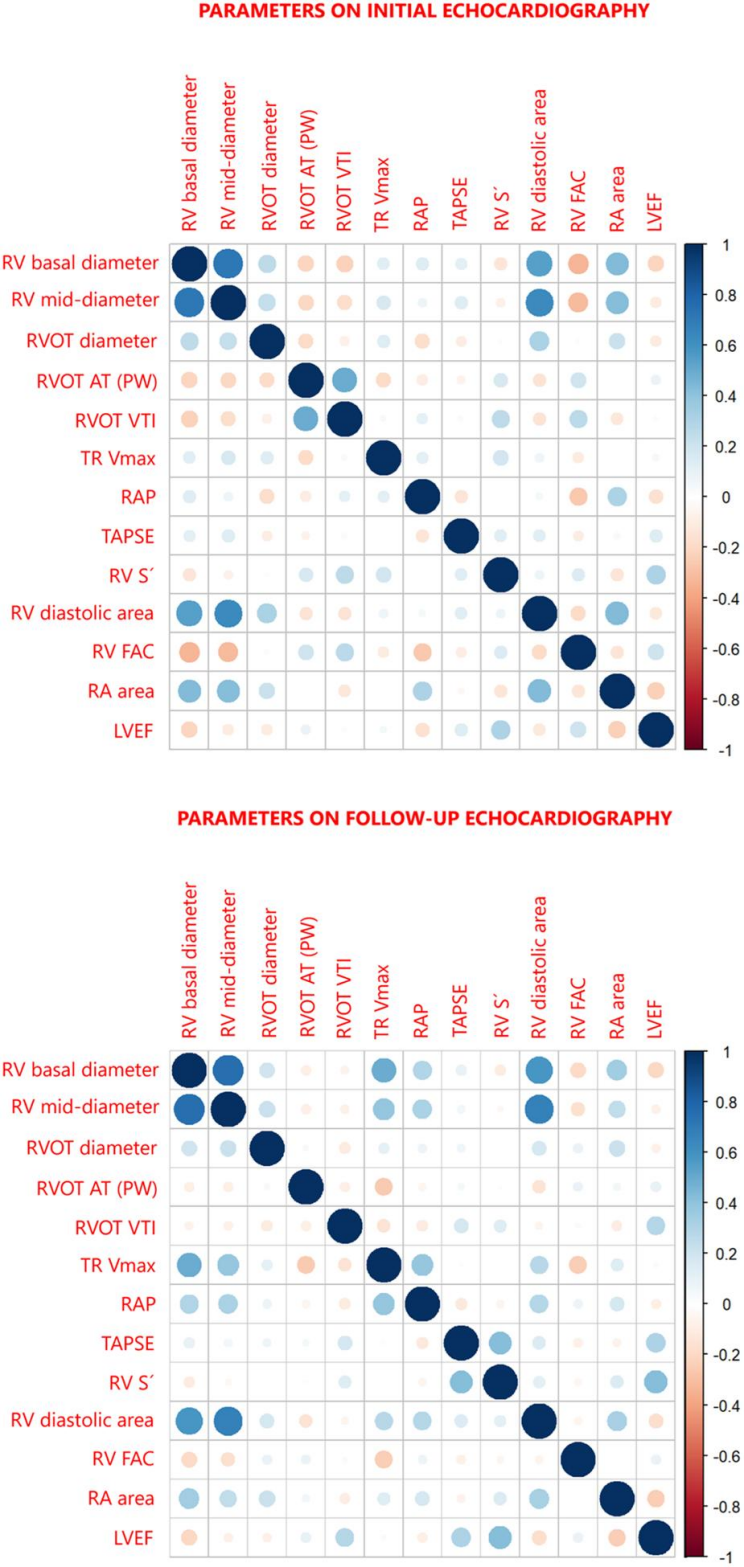
Correlation plots of echocardiographic parameters on pre- and post-echocardiography scans. *AT*, Acceleration time; *FAC*, fractional area change; *LVEF*, left ventricular ejection fraction; *PW*, pulsed-wave Doppler; *RA*, right atrium; *RAP*, right atrial pressure; *RV*, right ventricle; *RVOT*, right ventricular outflow tract; *TAPSE*, tricuspid annular plane systolic excursion; *TR*, tricuspid regurgitation; *V_max_*, maximum velocity; *VTI*, velocity time integral (stroke distance).

**Figure 4:**
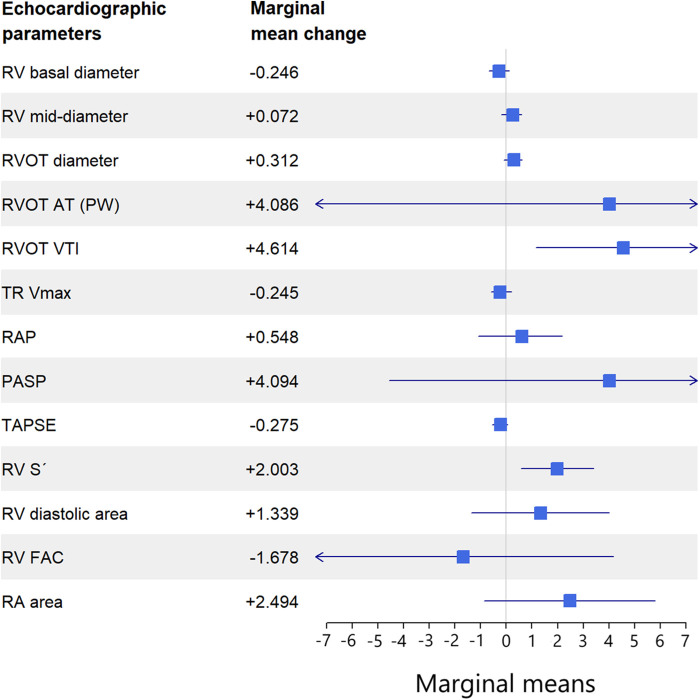
Forest plot depicting the marginal means of change in echocardiographic parameters on follow-up echocardiography among target and control groups. Non-parametric propensity score matching was performed with the target group consisting of patients who received any advanced reperfusion therapy, while the control group consisted of patients who did not receive any such therapy. *AT*, Acceleration time; *FAC*, fractional area change; *PASP*, pulmonary artery systolic pressure; *PW*, pulsed-wave Doppler; *RA*, right atrium; *RAP*, right atrial pressure; *RV*, right ventricle; *RVOT*, right ventricular outflow tract; *Ś*, lateral tricuspid annulus peak systolic velocity (on tissue Doppler); *TAPSE*, tricuspid annular plane systolic excursion; *TR*, tricuspid regurgitation; *V*_max_, maximum velocity; *VTI*, velocity time integral.

**Figure 5:**
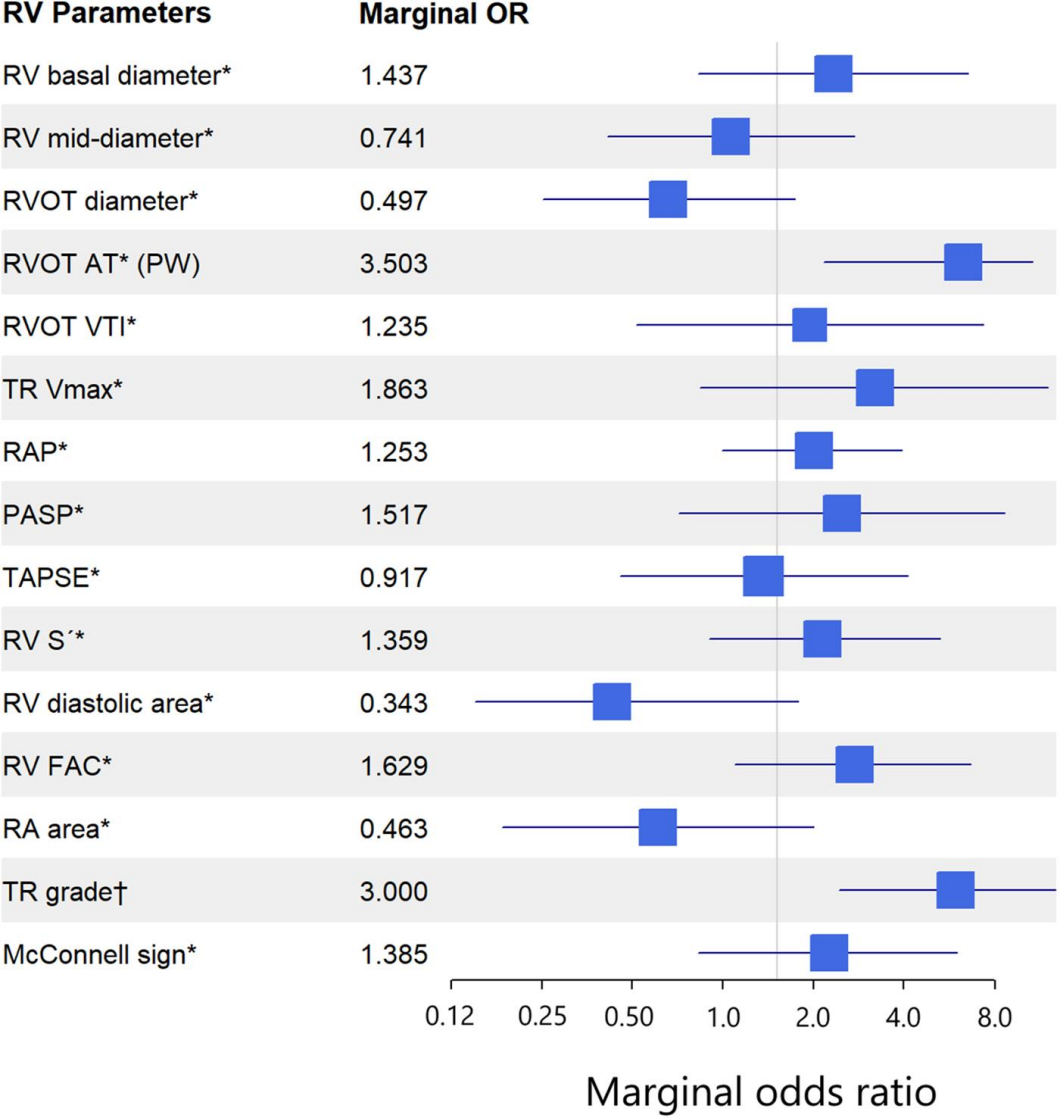
Forest plot depicting the marginal odds ratios for normalization of echocardiographic parameters on follow-up echocardiography between the two groups. Non-parametric propensity score matching was performed with the target group consisting of patients who received any advanced reperfusion therapy, while the control group consisted of patients who did not receive any such therapy. *AT*, Acceleration time; *FAC*, fractional area change; *FVE*, flow velocity envelope; *OR*, odds ratio; *PASP*, pulmonary artery systolic pressure; *PW*, pulsed-wave Doppler; *RA*, right atrium; *RAP*, right atrial pressure; *RV*, right ventricle; *RVOT*, right ventricular outflow tract; *Ś*, lateral tricuspid annulus peak systolic velocity (on tissue Doppler); *TAPSE*, tricuspid annular plane systolic excursion; *TR*, tricuspid regurgitation; *V*_max_, maximum velocity; *VTI*, velocity time integral. * Marginal odds for normalization of echocardiographic parameter; † marginal odds for improvement in TR grade.

**Figure 6:**
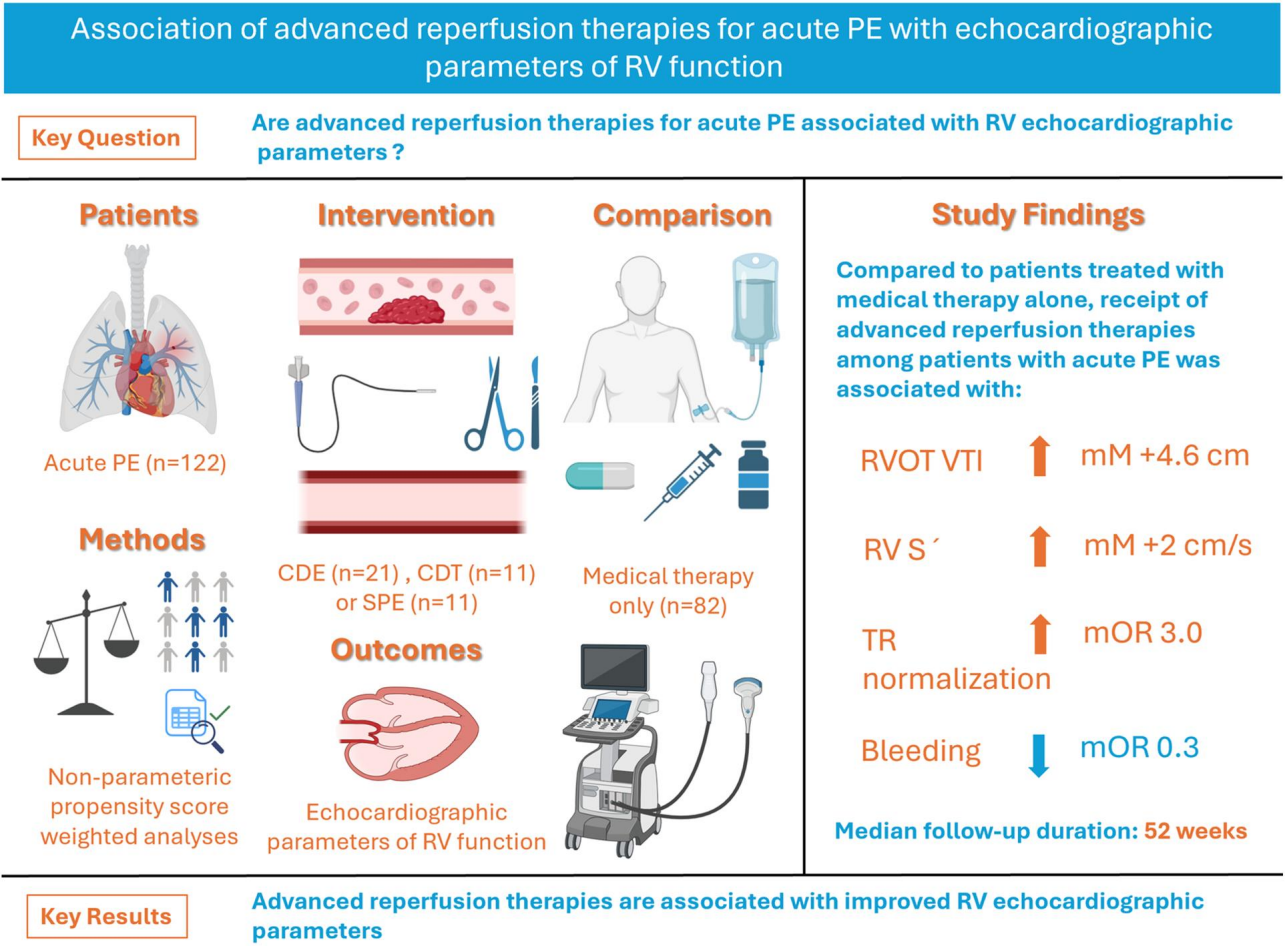
Visual summary. In this propensity score-matched retrospective analysis of 122 patients with acute pulmonary embolism, receipt of any advanced reperfusion therapy (CDT, CDE or SPE) was associated with statistically significant improvements in echocardiographic parameters of RV function as well as lower odds of 30-day major bleeding. *CDE*, catheter-directed embolectomy; *CDT*, catheter-directed thrombolysis; *mM*, marginal mean; *mOR*, marginal odds ratio; *PE*, pulmonary embolism; *TR*, tricuspid regurgitation; *RV*, right ventricle; *RVOT*, right ventricular outflow tract; *SPE*, surgical pulmonary embolectomy; *VTI*, velocity time integral.

The median values of echocardiographic parameters on initial (“pre”) and follow-up (“post”) studies in the propensity score-matched sample are provided in Table 5. Figure 3 depicts the correlation plot of linear relationships amongst the echocardiographic parameters on initial and follow-up studies. Patients who received any ART (CDE, CDT or SPE) had a 4.6 (95% CI: 1.2‒8.1) cm increase in RVOT VTI i.e., stroke distance (*p* = 0.009) and a 2.0 (95% CI: 0.6‒3.4) cm/s increase in RV Ś i.e., lateral tricuspid annulus peak systolic velocity (*p* = 0.006) as compared to the control group (details in Figure 4). Likewise, the odds of normalization of RVOT AT on pulsed-wave Doppler (marginal OR: 3.503; *p* < 0.001) and improvement in TR grade (marginal OR: 3.0; *p* = 0.002) were higher among patients who received any type of ART as compared to the control group (details in Figure 5). In terms of safety, patients who received any ART had lower 30-day major bleeding (marginal OR: 0.299; *p* < 0.001) as compared to patients who did not receive any ART. However, hospital LOS (marginal mean: +1.18 days; *p* = 0.584) did not differ significantly between the group of patients who received ART *vs.* those who did not receive any ART (see Figure 6).

**Table 5:** Echocardiographic parameters for the advanced therapy and control groups in the propensity score-matched sample (*n* = 122).

Quantitative parameters		Group	PRE		POST		mM^a^	*p* ^b^	
			Median* (IQR)		Median* (IQR)				
RV basal diameter (cm)		Intervention	4.46 (3.86‒4.96)		3.55 (3.34‒3.68)		−0.246	0.195	
		Control	4.50 (3.86‒4.85)		3.66 (3.31‒4.41)				
RV mid-diameter (cm)		Intervention	3.45 (2.93‒3.85)		2.73 (2.39‒3.02)		+0.072	0.682	
		Control	3.37 (2.93‒3.97)		2.90 (2.34‒3.23)				
RVOT diameter (cm)		Intervention	2.32 (1.93‒2.82)		2.54 (2.23‒2.64)		+0.312	0.052	
		Control	2.55 (2.19‒2.93)		2.38 (2.08‒2.70)				
RVOT PW AT (msec)		Intervention	69.4 (50.7‒80.5)		100.2 (91.9‒105.6)		+4.086	0.802	
		Control	71.0 (50.8‒86.8)		87.1 (59.5‒107.0)				
RVOT VTI (cm)		Intervention	11.1 (8.5‒11.1)		14.3 (11.3‒16.9)		**+4.614**	**0.009**	
		Control	10.3 (7.6‒12.1)		13.5 (10.9‒19.4)				
TR jet V_max_ (m/s)		Intervention	3.0 (2.5‒3.6)		2.1 (1.8‒2.3)		−0.245	0.176	
		Control	3.0 (2.5‒3.4)		2.6 (2.2‒3.1)				
RAP (mm Hg)		Intervention	3.5 (2.3‒4.7)		5 (5‒5)		+0.548	0.515	
		Control	5.0 (5.0‒10.2)		2.9 (1.4‒4.4)				
PASP (mm Hg)		Intervention	41.0 (31.2‒54.7)		25.2 (20.4‒27.0)		+4.094	0.356	
		Control	43.6 (32.8‒53.6)		35.4 (27.3‒48.6)				
TAPSE (cm)		Intervention	1.7 (1.5‒2.7)		2.2 (1.7‒2.4)		−0.275	0.078	
		Control	1.7 (1.2‒2.1)		2.0 (1.8‒2.4)				
RV Ś (cm/s)		Intervention	9.8 (7.7‒10.0)		11.9 (10.4‒13.4)		**+2.003**	**0.006**	
		Control	11.4 (8.5‒14.3)		11.5 (9.9‒13.9)				
RV diastolic area (cm^2^)		Intervention	22.2 (21.1‒26.9)		17.4 (14.2‒20.6)		+1.339	0.334	
		Control	24.8 (20.8‒30.2)		19.5 (15.4‒23.8)				
RV FAC (%)		Intervention	30.1 (26.1‒40.1)		36.7 (29.8‒41.0)		−1.678	0.577	
		Control	30.4 (21.4‒36.8)		35.5 (28.9‒42.9)				
RA Area (cm^2^)		Intervention	20.8 (14.2‒21.1)		17.4 (16.2‒21.0)		+2.494	0.146	
		Control	17.0 (14.4‒21.2)		15.3 (12.2‒17.8)				
**QUALITATIVE PARAMETERS**		**GROUP**	**PRE**		**POST**		**mOR** ^c^		***p*** ^b^
			**N** *	**%**	**N** *	**%**			
McConnell sign		Intervention	28.5	71.3%	0.4	0.9%	1.385		0.443
		Control	41.5	49.9%	5.9	7.1%			
TR grade	None	Intervention	4.2	11.6%	6.0	16.2%	**3.000**		**0.002**
		Control	7.9	13.0%	18.0	28.5%			
	Mild	Intervention	25.2	68.8%	31.0	83.8%			
		Control	35.4	58.3%	35.1	55.6%			
	Moderate	Intervention	7.2	19.7%	0.0	0.0%			
		Control	12.1	19.9%	5.4	8.6%			
	Severe	Intervention	0.0	0.0%	0.0	0.0%			
		Control	5.3	8.8%	4.6	7.3%			

* Weighted medians and frequencies computed in the propensity score-matched sample.

a Marginal mean of *Δ* (echocardiographic parameter on follow-up echocardiography subtracted from echocardiographic parameter on baseline echocardiography) based on linear regression models (in the propensity score-matched sample) exploring the association of *Δ* with the grouping variable (administration of advanced therapies) incorporating matching variables as interaction terms for a doubly robust estimate.

b *p*-values computed from multivariable regression models fitted in the propensity score-matched sample incorporating matching variables as interaction terms; *p*-values were adjusted for multiple comparisons using the modified Bonferroni correction method described by Hochberg (23).

c Marginal odds ratio computed from quasi-binomial regression models (in the propensity score-matched sample) exploring the association of outcome variable with the grouping variable (administration of advanced therapies) incorporating matching variables as interaction terms for a doubly robust estimate.

AT, Acceleration time; FAC, fractional area change; IQR, interquartile range; mM, marginal mean; mOR, marginal odds ratio; PASP, pulmonary artery systolic pressure; PW, pulse-wave Doppler; RA, right atrium; RAP, RA pressure; RV, right ventricle; RVOT, right ventricular outflow tract; TAPSE, tricuspid annular plane systolic excursion; TR, tricuspid regurgitation; V_max_, maximum velocity.

Statistically significant differences are highlighted in bold text.

We performed additional multiple propensity score matched analyses to assess the impact of receipt of individual therapies (CDE, CDT or SPE) on echocardiographic parameters of RV function as well as other outcomes. The results of these analyses were largely concordant with those of the pooled analysis mentioned previously. The details of these analyses are provided in the Supplementary Appendix (Supplementary Tables S1–S3 and Supplementary Figures S1–S9).

## Discussion

In this study of adult patients with acute PE, receipt of any type of ART (CDE, CDT or SPE) was associated with statistically significant improvements in echocardiographic parameters of RV function, that included higher odds of improvement in TR grade and normalization of RVOT AT on pulsed-wave Doppler as well as statistically significant increases in RV stroke distance and lateral tricuspid annulus peak systolic velocity as compared to those who did not receive any ART. Moreover, receipt of any type of ART (CDE, CDT or SPE) was also associated with reduced odds of 30-day major bleeding (marginal OR: 0.299; *p* < 0.001) as compared to patients who did not receive any ART. Additionally, hospital LOS was not significantly different between the group of patients who received ART vs. those who were treated with therapeutic anticoagulation alone. Given the modest sample size, non-randomized design, and potential for residual confounding despite propensity score matching, these findings should be interpreted as hypothesis-generating rather than definitive evidence of treatment efficacy.

The optimal treatment of intermediate- to high-risk acute PE remains incompletely defined (11). Percutaneous interventions, such as CDT and CDE, were designed with the aim of affording the hemodynamic benefits of ST on RV function without the concomitant potential increase in hemorrhagic complications (10, 11). However, data regarding their efficacy and safety are mostly based on short-term observational studies (12, 13, 24). CDT and CDE is associated with stabilization of pulmonary artery hemodynamics, improvement in right heart strain and decrease in pulmonary hypertension (25), as well as a reduction in the Miller Index—a quantification of the severity of PE based on the degree of pulmonary arterial obstruction (26). Results of the US cohort of the FLASH registry demonstrated that among 800 patients with acute PE, CDE performed using the FlowTriever® (Inari Medical, Irvine, CA) device was associated with a 0.8% rate of 30-day mortality and 1.4% rate of major bleeding at 48 h (27). Results of the first 150 patients in the STRIKE-PE study showed that CDE performed using the Indigo® aspiration system (Penumbra, Alameda, CA) was associated with significant reductions in PASP at 48 h and Borg dyspnea scale score at 90 days along with a 2.7% rate of major adverse events (28). Moreover, results of the STORM-PE trial demonstrated that CDE for intermediate high-risk PE resulted in greater improvements in RV/LV ratio without a concomitant increase in 7-day major adverse events (14). The PEERLESS II randomized controlled trial, comparing percutaneous catheter interventions for intermediate- to high-risk acute PE vs. systemic anticoagulation alone, is currently enrolling patients and is anticipated to complete enrollment in late 2026 (29). Most recently, HI-PEITHO provided the strongest randomized clinical-outcome evidence to date for a catheter-based strategy in selected intermediate-risk PE: ultrasound-facilitated CDT plus anticoagulation reduced the 7-day composite of PE-related death, cardiorespiratory decompensation or collapse, or symptomatic PE recurrence compared with anticoagulation alone (15, 30). Importantly, this benefit was primarily driven by reduced early clinical deterioration rather than a demonstrated mortality difference, and longer-term functional and quality-of-life outcomes remain under follow-up. PRAGUE-26 is also ongoing and will test whether CDT improves clinical outcomes compared with anticoagulation alone in intermediate-high-risk PE, with planned assessment of bleeding, first-line therapy failure, cost-effectiveness, and functional/patient-reported outcomes over 2 years (16). These trials provide important clinical context for our findings, but they do not eliminate the need for studies focused specifically on intermediate-term RV recovery after different reperfusion strategies (24, 31, 32). In a recent retrospective study of 113 patients with intermediate or high-risk PE, recovery of long- term RV function was more likely in patients receiving advanced catheter directed therapies or systemic thrombolysis compared to anticoagulation alone (33). Our study provides observational evidence that ART in patients with intermediate- to high-risk acute PE afford statistically significant improvements in echocardiographic parameters of RV function, without an increase in adverse outcomes.

Our findings should also be interpreted in the context of the marked heterogeneity of VTE and PE. Patients with PE differ not only by conventional risk category, but also by thrombus location, clot burden, RV reserve, comorbidity profile, bleeding risk, provoking factors, and baseline functional status. Registry studies have demonstrated that VTE outcomes vary substantially in special populations, including patients with rare vascular disorders such as hereditary hemorrhagic telangiectasia, supporting the broader principle that VTE is not a uniform disease process (34). Similarly, Fernández-Capitán and colleagues showed that outcomes differ between symptomatic subsegmental PE and more central PE during anticoagulation, emphasizing that anatomical extent and clot distribution may influence prognosis and treatment decisions (35). In the present cohort, most patients had central PE and imaging evidence of right heart strain, which may represent a population more likely to demonstrate measurable RV recovery after reperfusion therapy than patients with smaller or more peripheral clot burden.

Transthoracic echocardiography remains the most common modality for assessment of RV function despite the challenges entailed in making an accurate assessment of RV function by echocardiography (21, 36). RV assessment by echocardiography is complicated by the complex geometry of the RV, a unique pattern of contraction, and the fact that RV echocardiographic parameters are greatly influenced by volume status and respiratory effort (36). Moreover, measurements of echocardiographic parameters can be influenced by probe angulation and the quality of echocardiographic windows (21). Despite this, echocardiography remains the most common non-invasive modality for assessment of RV function (37). The American Society of Echocardiography and the British Society of Echocardiography have provided guidance for standardizing the measurement of echocardiographic parameters of RV function (21, 36). In the present study, each echocardiography study was re-interpreted in a structured and blinded manner to ensure that echocardiographic parameters were recorded in a standardized fashion and bias resulting from interobserver variability and measurement errors was minimized. Moreover, multiple echocardiographic parameters of RV function were analyzed to ensure that assessment of overall RV function was robust. The fact that we observed improvement in multiple RV echocardiographic parameters in the ART group in both qualitative and quantitative analyses reflects that these findings were robust. Improvements in RVOT VTI and RV Ś are key indicators of enhanced RV systolic performance. RVOT VTI (stroke distance) is a surrogate marker for RV stroke volume, while RV Ś reflects the longitudinal RV systolic function (37). Additionally, the normalization of AT on pulsed-wave Doppler interrogation of the RVOT signifies a reduction in pulmonary vascular resistance indicative of improved pulmonary hemodynamics (38). Furthermore, the improvement in TR grade likely reflects a combination of better right heart hemodynamics and evidence of right ventricular reverse remodeling (36–38), highlighting a multifaceted response to therapeutic intervention. Although improvement in RVOT VTI, RV Ś, RVOT AT, and TR grade suggests favorable RV-pulmonary vascular recovery, these echocardiographic parameters remain surrogate endpoints. Their clinical importance lies in their potential relationship to exercise capacity, post-PE syndrome, recurrent healthcare utilization, and longer-term cardiopulmonary prognosis. The present study was not powered to determine whether echocardiographic improvement translated into lower mortality or fewer late complications. Real-world VTE studies have demonstrated that treatment-related exposures may be associated with mortality differences during anticoagulation, as illustrated by Siniscalchi and colleagues in the RIETE registry (39). Therefore, future PE reperfusion studies should ideally pair serial RV imaging with patient-centered outcomes, functional testing, recurrent VTE, bleeding, mortality, and post-PE syndrome measures.

In the absence of randomized controlled trials, observational studies and real-world data provide the best surrogate evidence for efficacy and safety of ART for acute PE. Selection and confounding bias are important considerations in observational studies (40). Non-parametric propensity score matching is a valid statistical technique that helps to control for important confounders in observational studies while making minimal assumptions about the underlying population (22). In the present study, propensity score matching was used to control the influence of PESI score, ESC risk group, CPES score, BMI and age on the association between receipt of ART and RV echocardiographic parameters. Moreover, we included a consecutive sample of patients treated by PERT in a single hospital system to minimize the risk of selection bias. We found that receipt of any ART among patients with acute PE was significantly associated with improvement in RV stroke distance and lateral tricuspid annulus peak systolic velocity as well as higher odds of normalization of RVOT AT and improvement in TR grade at a median follow-up of 26.6 weeks. In another study of 150 patients, Goldberg and colleagues reported that SPE for intermediate high- and high-risk acute PE provided immediate improvements in RV function in the post-operative period as assessed by RV/LV ratio and RV FAC on echocardiography (41). The results of our study are concordant and plausible when keeping in view the additional recent findings reported by other investigators (27, 28, 32, 33, 41, 42). The epidemiology of VTE is also dynamic, as illustrated by multinational registry analyses of venous thrombosis occurring after SARS-CoV-2 vaccination (43), which reinforces the need for adaptable risk stratification and treatment pathways as new prothrombotic clinical contexts emerge. In another retrospective cohort study of 6,746 hospitalizations for submassive PE, Semaan et al. reported that CDT plus anticoagulation afforded better survival at 6 months and similar rate of bleeding complications when compared to the anticoagulation alone group (32). In a meta-analysis comparing CDT vs. systemic anticoagulation alone for submassive PE, CDT was associated with lower in-hospital, 30-day and 90-day mortality as well as similar bleeding rates compared with systemic anticoagulation alone (44). While we await the completion of larger, well-powered, randomized trials to provide evidence regarding hard clinical endpoints, data from observational studies including the present study provide the best surrogate evidence reflecting the favorable impacts of ART on short-term and intermediate-term RV function among patients with acute PE.

Several limitations should be considered. First, this was a retrospective, non-randomized study from a single health system with a modest sample size, and the effective sample size after propensity score weighting was smaller than the original cohort. Therefore, the study may have been underpowered for some outcomes and should be considered hypothesis-generating. Second, treatment selection was determined by PERT-based clinical decision-making rather than random allocation. Although propensity score matching was used to improve covariate balance, selection bias, confounding by indication, and residual/unmeasured confounding remain possible, particularly because anatomic clot burden, RV reserve, bleeding risk, clinician judgment, and procedural availability may have influenced treatment selection. Third, echocardiographic parameters are clinically relevant but imperfect surrogate endpoints; this study was not designed to determine whether improvements in RV function translated into lower mortality, fewer recurrent PE events, improved exercise capacity, or reduced post-PE syndrome. Fourth, all echocardiographic studies were reinterpreted by a single blinded observer, which improved internal consistency but precluded assessment of interobserver reliability. Fifth, RV longitudinal strain, Tei index, cardiopulmonary exercise testing, quality-of-life measures, and right heart catheterization data were not systematically available. Finally, all patients were managed within a structured PERT program in an urban academic health system, and the findings may not be generalizable to institutions without comparable multidisciplinary PE expertise or procedural resources.

## Conclusion

Among adult patients with acute PE, receipt of any advanced reperfusion therapy (catheter-directed embolectomy, catheter-directed thrombolysis or surgical pulmonary embolectomy) was associated with statistically significant increases in RV stroke distance and RV Ś (lateral tricuspid annulus peak systolic velocity) as well as higher odds of improvement of TR grade as compared to patients, matched for PE severity, who did not receive any such reperfusion therapy. Advanced reperfusion therapies were also associated with lower odds of 30-day major bleeding without any adverse association with hospital LOS. Given the modest sample size, non-randomized design, and potential for residual confounding despite propensity score matching, these findings should be interpreted as hypothesis-generating, rather than definitive evidence of treatment efficacy. Larger prospective studies and randomized trials incorporating serial RV imaging, functional status, post-PE syndrome, bleeding, recurrent VTE, and mortality are needed to clarify which patients derive durable clinical benefit from advanced reperfusion strategies.

## Data Availability

The raw data supporting the conclusions of this article will be made available by the authors, without undue reservation.
