# Supplementary material for: Association of advanced therapies for intermediate- to high-risk pulmonary embolism with improved right ventricular function on outpatient follow-up among survivors
Source: Front Cardiovasc Med. 2026 Jun 10;13:1767184. doi: 10.3389/fcvm.2026.1767184 (PMC13290519; doi:10.3389/fcvm.2026.1767184)
Supplement: Supplementary file 1 [file Presentation1.pdf]

Supplementary Information

This file contains supplementary methods and results for the original article "Association of advanced therapies for intermediate- to high-risk pulmonary embolism with improved right ventricular function on outpatient follow-up among survivors" by Sheikh *et al.*

Propensity score-matched analysis for catheter-directed embolectomy (CDE)

For investigating the association between echocardiographic parameters and CDE, a propensity score-matched analysis was performed. Patients who had received CDE were designated as the target group and patients in the control group (those who had not received *any* advanced therapy) were matched on the propensity score to one or more patients in the target group using the optimal *full* matching specification—with the desired estimand being the average treatment effect ("ATE") in the population. The effective sample size of the propensity score-weighted sample was 71.5. Love plot showed improved balance overall in the matched sample (Supplementary Figure S1).

The median values of echocardiographic parameters on initial ("pre") and follow-up ("post") studies in the propensity score-matched sample are provided in Supplementary Table S1. Patients who received CDE had significant improvement in echocardiographic parameters, both on quantitative analyses (Supplementary Figure S2) and qualitative analyses (Supplementary Figure S3). Most notably, patients who underwent CDE had higher odds of normalization of RVOT AT (marginal OR: 4.217; p<0.001), PASP (marginal OR: 4.049; p=0.005) and RV S′ (marginal OR: 4.031; p<0.001) compared to the control group.

In terms of clinical outcomes, patients who underwent CDE had lower odds of 30-day major bleeding (marginal OR: 0.607; p<0.001) as compared to patients who did not receive any

ART. However, hospital LOS was significantly longer (marginal mean: +3.300; $p<0.001$) for patients who underwent CDE *vs.* those who did not undergo CDE.

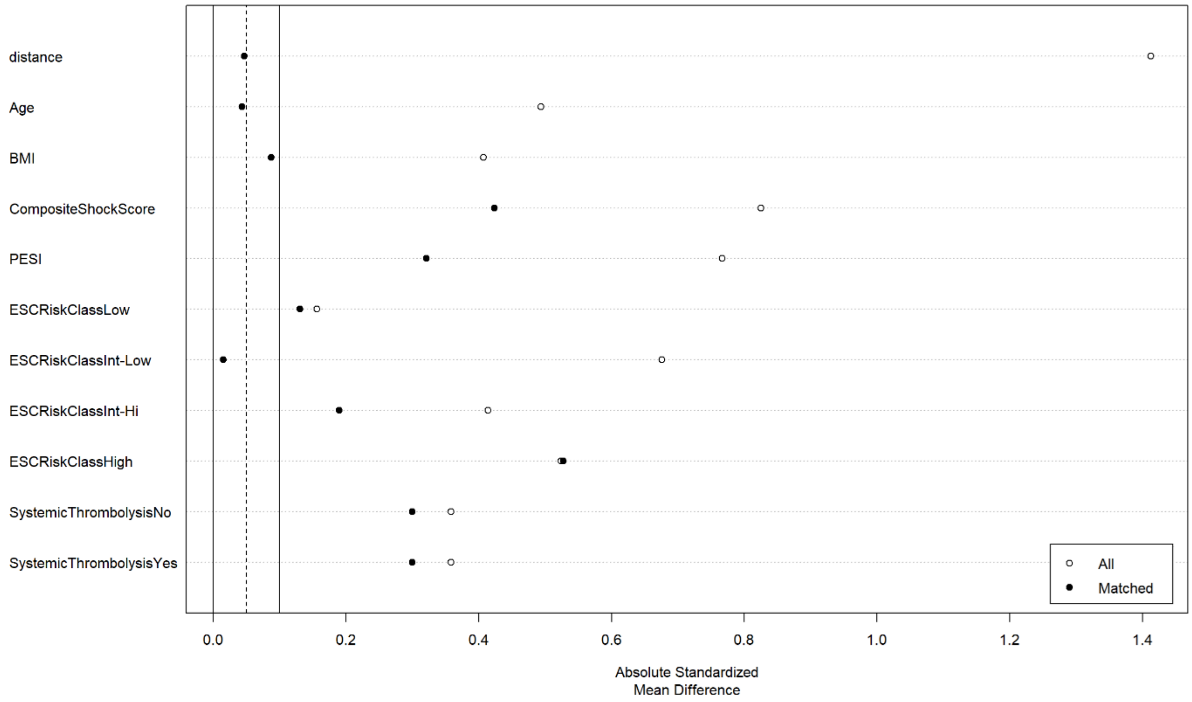

Supplementary Figure S1: Love plot depicting the absolute standardized mean differences on the matching variables (age, BMI, PESI score, ESC risk group, Composite PE Shock score and systemic thrombolysis) between the target (patients who received CDE) and the control (patients who did not receive any advanced therapy) groups. *BMI*=Body mass index; *CDE*=catheter-directed embolectomy; *ESC*=European Society of Cardiology; *PE*=pulmonary embolism; *PESI*=PE Severity Index.

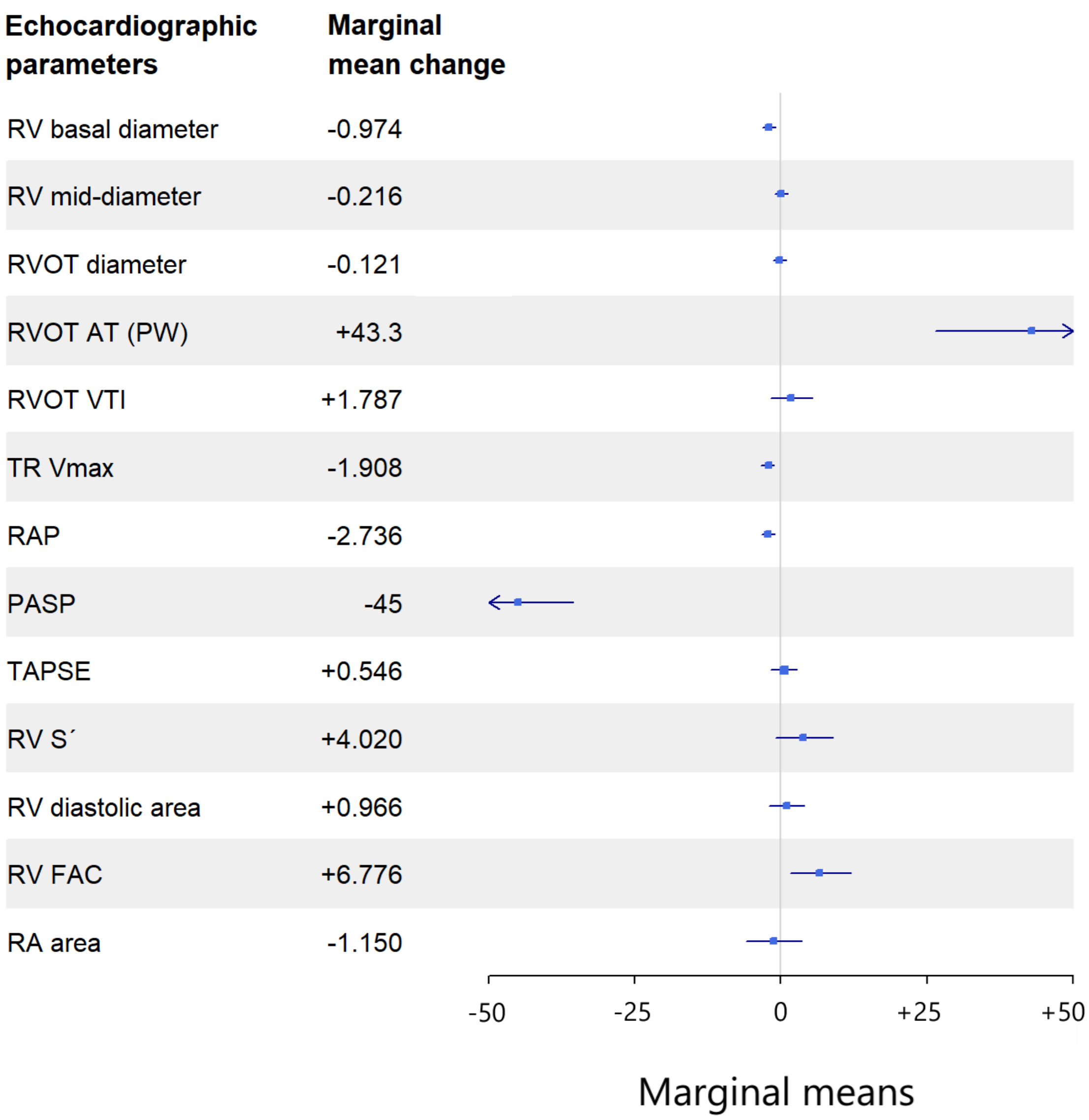

Supplementary Figure S2: Forest plot depicting the marginal means of change in echocardiographic parameters on follow-up echocardiography among patients who received CDE *vs.* those who did not receive any advanced therapy in the propensity score-matched sample. *AT*=Acceleration time; *FAC*=fractional area change; *PASP*=pulmonary artery systolic pressure; *PW*=pulsed-wave Doppler; *RA*=right atrium; *RAP*=right atrial pressure; *RV*=right ventricle; *RVOT*=right ventricular outflow tract; *S´*=lateral tricuspid annulus peak

systolic velocity (on tissue Doppler); *TAPSE*=tricuspid annular plane systolic excursion; *TR*=tricuspid regurgitation; V_{max}=maximum velocity; *VTI*=velocity time integral.

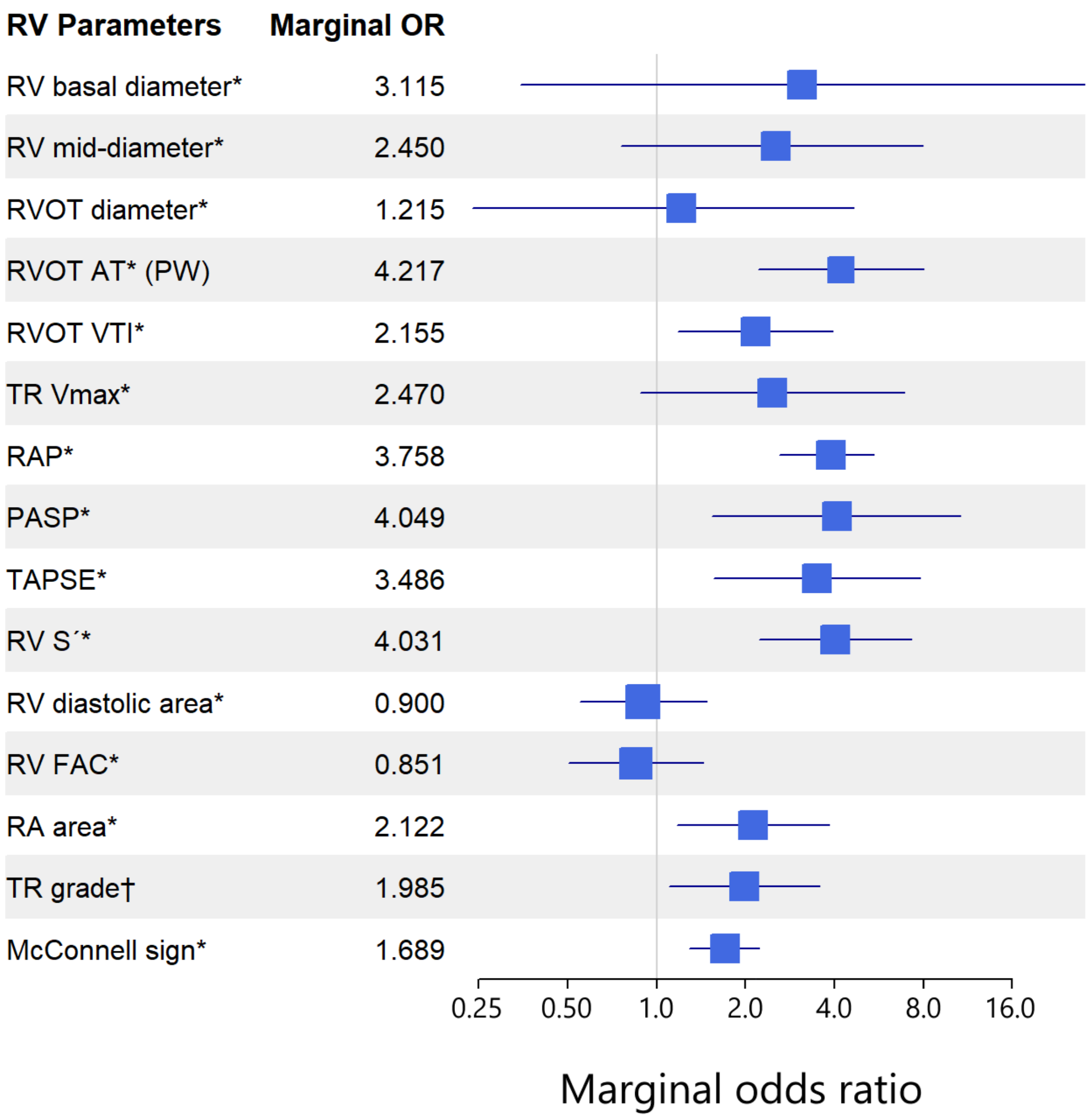

Supplementary Figure S3: Forest plot depicting the marginal odds ratios for normalization of various echocardiographic parameters on follow-up echocardiography among patients who received CDE *vs.* those who did not receive any advanced therapy in the propensity

score-matched sample. *AT*=Acceleration time; *FAC*=fractional area change; *FVE*=flow velocity envelope; *OR*=odds ratio; *PASP*=pulmonary artery systolic pressure; *PW*=pulsed-wave Doppler; *RA*=right atrium; *RAP*=right atrial pressure; *RV*=right ventricle; *RVOT*=right ventricular outflow tract; *S´*=lateral tricuspid annulus peak systolic velocity (on tissue Doppler); *TAPSE*=tricuspid annular plane systolic excursion; *TR*=tricuspid regurgitation; V_{max}=maximum velocity; *VTI*=velocity time integral. * Marginal odds for normalization of echocardiographic parameter; † marginal odds for improvement in TR grade.

Supplementary Table S1: Echocardiographic parameters for the intervention (catheter-directed embolectomy) and control groups in the propensity score-matched sample (n=122)

QUANTITATIVE PARAMETERS	GROUP	PRE	POST	mM†	*p*‡
		Median* (IQR)	Median* (IQR)		
RV basal diameter (cm)	Intervention	4.93 (4.54–4.99)	3.25 (3.13–3.70)	**-0.974**	**<0.001**
	Control	4.47 (3.86–4.86)	3.69 (3.29–4.42)		
RV mid-diameter (cm)	Intervention	3.63 (3.00–3.89)	2.37 (2.31–3.19)	-0.216	0.202
	Control	3.37 (2.92–4.03)	2.91 (2.32–3.23)		
RVOT diameter (cm)	Intervention	2.38 (2.20–3.14)	2.62 (2.47–2.67)	-0.121	0.420
	Control	2.53 (2.15–2.93)	2.38 (2.10–2.76)		
RVOT PW AT (msec)	Intervention	47.6 (45.5–66.4)	116.2 (95.2–118.2)	**+43.3**	**<0.001**
	Control	71.8 (53.5–88.0)	84.0 (59.4–101.8)		
RVOT VTI (cm)	Intervention	5.7 (5.0–8.9)	12.3 (10.8–13.9)	+1.787	0.325
	Control	10.5 (7.7–15.1)	13.4 (10.8–16.8)		
TR jet V_{max} (m/s)	Intervention	3.0 (2.5–3.5)	2.1 (2.3–2.4)	**-1.908**	**<0.001**
	Control	2.6 (2.2–3.2)	2.5 (2.2–2.8)		
RAP (mm Hg)	Intervention	5.0 (5.0–7.9)	2.9 (1.4–4.4)	**-2.736**	**0.002**
	Control	5.0 (5.0–10.5)	5.0 (5.0–5.0)		
PASP (mm Hg)	Intervention	33.2 (24.2–51.0)	27.3 (26.5–28.0)	**-45**	**<0.001**
	Control	44.0 (33.2–56.7)	36.2 (27.4–50.4)		
TAPSE (cm)	Intervention	1.7 (1.5–2.2)	2.4 (2.3–2.5)	+0.546	0.634
	Control	1.8 (1.3–2.1)	2.0 (1.8–2.4)		
RV S´ (cm/s)	Intervention	9.1 (5.8–13.6)	12.4 (11.7–13.1)	+4.020	0.111
	Control	11.6 (8.6–14.6)	11.4 (9.9–14.0)		
RV diastolic area (cm^2)	Intervention	28.0 (22.0–29.2)	17.0 (16.7–19.1)	+0.966	0.535
	Control	24.4 (20.7–30.6)	20.0 (15.4–23.9)		

RV FAC (%)		Intervention	30.6 (21.3–36.8)		35.3 (28.8–42.9)		**+6.776**	**0.010**
		Control	30.1 (19.3–37.5)		31.2 (24.8–35.5)			
RA Area (cm^2)		Intervention	20.7 (19.1–20.9)		16.9 (11.6–19.3)		-1.150	0.634
		Control	17.0 (14.3–20.8)		15.0 (12.1–17.9)			
**QUALITATIVE PARAMETERS**		**GROUP**	**PRE**		**POST**		**mOR§**	***p*‡**
			**N***	**%**	**N***	**%**		
McConnell sign		Intervention	10.6	55.9%	0.0	0.0%	**1.689**	**<0.001**
		Control	43.1	52.0%	6.1	7.4%		
TR grade	None	Intervention	4.1	26.2%	4.7	26.8%	**1.985**	**0.022**
		Control	8.6	13.3%	18.9	28.1%		
	Mild	Intervention	11.3	72.3%	12.8	73.2%		
		Control	38.3	59.1%	37.7	56.0%		
	Moderate	Intervention	0.2	1.5%	0.0	0.0%		
		Control	11.4	17.6%	5.3	7.8%		
	Severe	Intervention	0.0	0.0%	0.0	0.0%		
		Control	6.4	9.9%	5.4	8.1%		

* Weighted medians and frequencies computed in the propensity score-matched sample

† Marginal mean of Δ (echocardiographic parameter on follow-up echocardiography subtracted from echocardiographic parameter on baseline echocardiography) based on linear regression models (in the propensity score-matched sample) exploring the association of Δ with the grouping variable (administration of catheter-directed embolectomy) incorporating matching variables as interaction terms for a doubly robust estimate

‡ *p*-values computed from multivariable regression models fitted in the propensity score-matched sample incorporating matching variables as interaction terms; *p*-values were adjusted for multiple comparisons using the modified Bonferroni correction method described by Hochberg (1988)

§ Marginal odds ratio computed from quasi-binomial regression models (in the propensity score-matched sample) exploring the association of outcome variable with the grouping variable (administration of catheter-directed embolectomy) incorporating matching variables as interaction terms for a doubly robust estimate

AT=Acceleration time; *FAC*=fractional area change; *IQR*=interquartile range; *mM*=marginal mean; *mOR*=marginal odds ratio; *PASP*=pulmonary artery systolic pressure; *PW*=pulse-wave Doppler; *RA*=right atrium; *RAP*=RA pressure; *RV*=right ventricle; *RVOT*=right ventricular

outflow tract; *TAPSE*=tricuspid annular plane systolic excursion; *TR*=tricuspid regurgitation; V_{max}=maximum velocity.

Propensity score-matched analysis for catheter-directed thrombolysis (CDT)

For investigating the association between echocardiographic parameters and CDT, a propensity score-matched analysis was performed. Patients who had received CDT were designated as the target group and patients in the control group (those who had not received *any* advanced therapy) were matched on the propensity score to one or more patients in the target group using the optimal *full* matching specification—with the desired estimand being the average treatment effect ("ATE") in the population. The effective sample size of the propensity score-weighted sample was 76.1. Love plot showed improved balance overall in the matched sample (Supplementary Figure S4).

The median values of echocardiographic parameters on initial ("pre") and follow-up ("post") studies in the propensity score-matched sample are provided in Supplementary Table S2. Patients who received CDT had significant improvement in echocardiographic parameters, both on quantitative analyses (Supplementary Figure S5) and qualitative analyses (Supplementary Figure S6). Most notably, patients who underwent CDT had higher odds of normalization of McConnell sign (marginal OR: 2.793; $p<0.001$) and TAPSE (marginal OR: 3.681; $p<0.001$) compared to the control group.

In terms of clinical outcomes, odds of 30-day major bleeding (marginal OR: 1.000; $p=0.998$) and hospital LOS (marginal mean: -3.752; $p=0.436$) did not differ significantly for patients who received CDT as compared to patients who did not receive any ART.

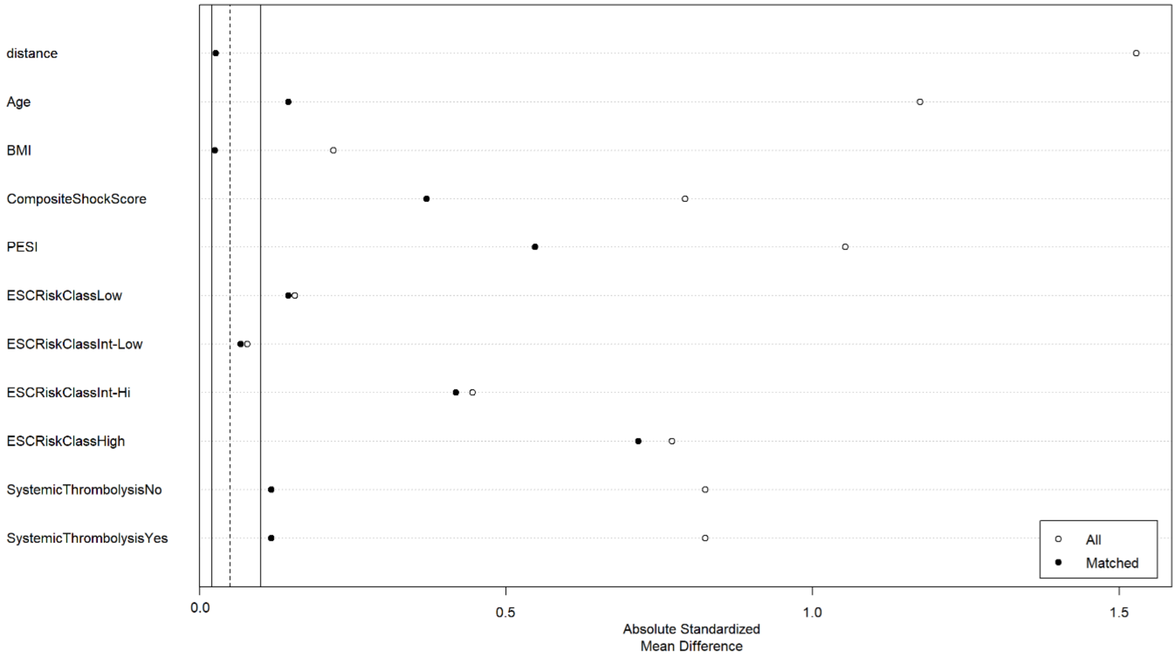

Supplementary Figure S4: Love plot depicting the absolute standardized mean differences on the matching variables (age, BMI, PESI score, ESC risk group, Composite PE Shock score and systemic thrombolysis) between the target (patients who received CDT) and the control (patients who did not receive any advanced therapy) groups. *BMI*=Body mass index; *CDE*=catheter-directed embolectomy; *ESC*=European Society of Cardiology; *PE*=pulmonary embolism; *PESI*=PE Severity Index.

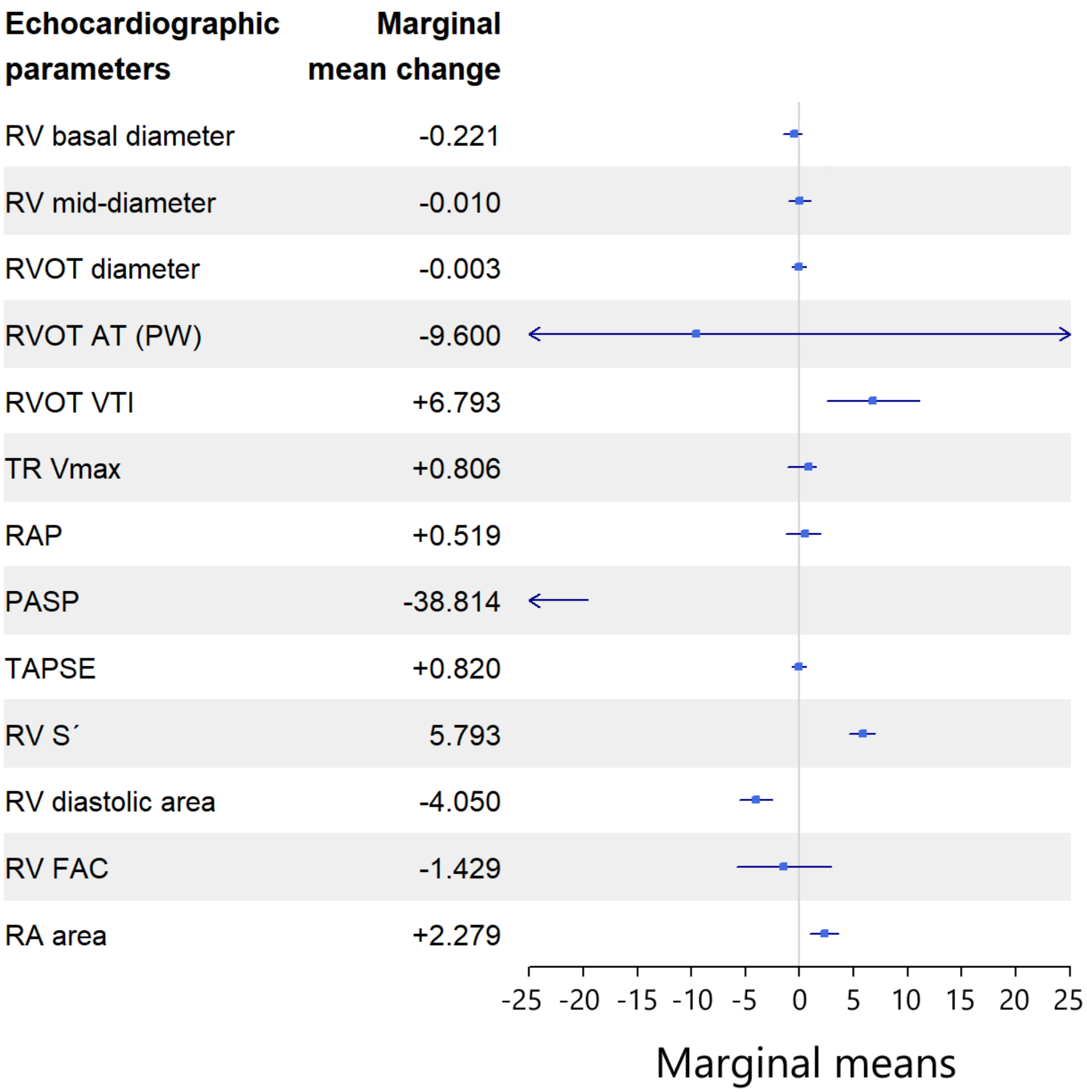

Supplementary Figure S5: Forest plot depicting the marginal means of change in echocardiographic parameters on follow-up echocardiography among patients who received CDT *vs.* those who did not receive any advanced therapy in the propensity score-matched sample. *AT*=Acceleration time; *FAC*=fractional area change; *PASP*=pulmonary artery systolic pressure; *PW*=pulsed-wave Doppler; *RA*=right atrium; *RAP*=right atrial pressure; *RV*=right ventricle; *RVOT*=right ventricular outflow tract; *S´*=lateral tricuspid annulus peak

systolic velocity (on tissue Doppler); *TAPSE*=tricuspid annular plane systolic excursion; *TR*=tricuspid regurgitation; V_{max}=maximum velocity; *VTI*=velocity time integral.

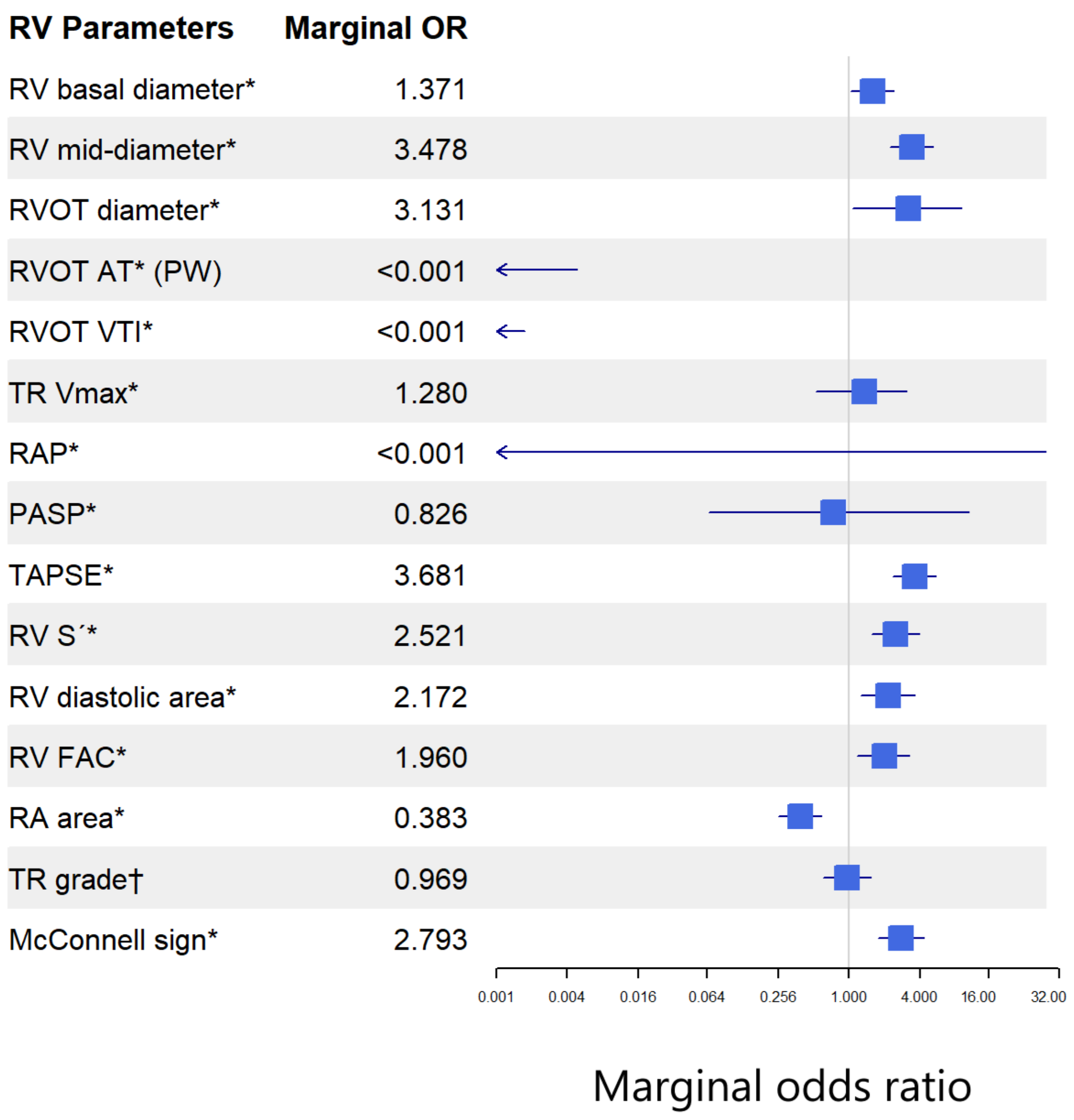

Supplementary Figure S6: Forest plot depicting the marginal odds ratios for normalization of various echocardiographic parameters on follow-up echocardiography among patients who received CDT *vs.* those who did not receive any advanced therapy in the propensity

score-matched sample. *AT*=Acceleration time; *FAC*=fractional area change; *FVE*=flow velocity envelope; *OR*=odds ratio; *PASP*=pulmonary artery systolic pressure; *PW*=pulsed-wave Doppler; *RA*=right atrium; *RAP*=right atrial pressure; *RV*=right ventricle; *RVOT*=right ventricular outflow tract; *S´*=lateral tricuspid annulus peak systolic velocity (on tissue Doppler); *TAPSE*=tricuspid annular plane systolic excursion; *TR*=tricuspid regurgitation; V_{max}=maximum velocity; *VTI*=velocity time integral. * Marginal odds for normalization of echocardiographic parameter; † marginal odds for improvement in TR grade.

Supplementary Table S2: Echocardiographic parameters for the intervention (catheter-directed thrombolysis) and control groups in the propensity score-matched sample (n=122)

QUANTITATIVE PARAMETERS	GROUP	PRE	POST	mM†	*p*‡
		Median* (IQR)	Median* (IQR)		
RV basal diameter (cm)	Intervention	4.17 (4.13–4.68)	3.37 (3.25–3.49)	-0.221	0.300
	Control	4.28 (3.75–4.79)	3.65 (3.25–4.30)		
RV mid-diameter (cm)	Intervention	3.20 (2.99–3.70)	2.40 (2.40–2.71)	-0.010	0.984
	Control	3.33 (2.87–3.78)	2.82 (2.29–3.23)		
RVOT diameter (cm)	Intervention	2.26 (2.10–2.34)	2.08 (2.00–2.20)	-0.003	0.987
	Control	2.50 (2.13–2.97)	2.40 (2.15–2.80)		
RVOT PW AT (msec)	Intervention	82.4 (78.6–88.3)	54.7 (52.5–113.0)	-9.600	0.222
	Control	72.8 (51.0–90.5)	77.8 (57.9–106.0)		
RVOT VTI (cm)	Intervention	7.64 (3.7–11.5)	11.5 (11.5–11.6)	**+6.793**	**0.002**
	Control	9.9 (7.4–14.0)	13.4 (10.8–16.7)		
TR jet V_{max} (m/s)	Intervention	2.1 (2.1–2.3)	1.2 (1.0–1.3)	+0.806	0.817
	Control	2.9 (2.4–3.5)	2.6 (2.2–3.2)		
RAP (mm Hg)	Intervention	5.0 (5.0–5.0)	5.0 (5.0–5.0)	+0.519	0.730
	Control	5.0 (5.0–10.7)	3.0 (1.4–4.5)		
PASP (mm Hg)	Intervention	22.6 (22.6–27.8)	10.8 (9.2–11.9)	-38.814	0.000
	Control	42.4 (31.7–56.5)	36.2 (26.9–49.2)		
TAPSE (cm)	Intervention	1.1 (1.3–1.6)	2.1 (2.0–2.4)	**+0.820**	**<0.001**
	Control	1.8 (1.3–2.1)	1.9 (1.7–2.3)		
RV S´ (cm/s)	Intervention	9.8 (9.8–9.9)	11.2 (11.0–11.5)	**+5.793**	**<0.001**
	Control	11.8 (8.9–14.5)	11.7 (9.9–14.0)		
RV diastolic area (cm^2)	Intervention	22.6 (20.7–26.8)	20.5 (19.2–21.1)	**-4.050**	**<0.001**
	Control	23.9 (20.0–29.3)	19.2 (15.0–23.9)		

RV FAC (%)		Intervention	31.0 (29.1–31.4)		38.9 (37.4–40.4)		-1.429	0.523
		Control	29.7 (21.0–36.8)		36.1 (29.3–43.5)			
RA Area (cm^2)		Intervention	19.6 (18.4–20.9)		16.0 (15.3–16.8)		**+2.279**	**<0.001**
		Control	17.1 (14.1–21.2)		15.7 (11.9–17.9)			
**QUALITATIVE PARAMETERS**		**GROUP**	**PRE**		**POST**		**mOR§**	***p*‡**
			**N***	**%**	**N***	**%**		
McConnell sign		Intervention	8	100%	0.1	1.5%	**2.793**	**<0.001**
		Control	39.7	61.9%	6.5	10.2%		
TR grade	None	Intervention	0.0	0.0%	0.2	3.2%	0.969	0.892
		Control	9.4	15.1%	19.8	30.4%		
	Mild	Intervention	7.7	100%	7.5	96.8%		
		Control	34.9	56.0%	34.0	52.3%		
	Moderate	Intervention	0.0	0.0%	0.0	0.0%		
		Control	11.3	18.2%	5.6	8.6%		
	Severe	Intervention	0.0	0.0%	0.0	0.0%		
		Control	6.7	10.7%	5.7	8.8%		

† Marginal mean of Δ (echocardiographic parameter on follow-up echocardiography subtracted from echocardiographic parameter on baseline echocardiography) based on linear regression models (in the propensity score-matched sample) exploring the association of Δ with the grouping variable (administration of catheter-directed thrombolysis) incorporating matching variables as interaction terms for a doubly robust estimate

‡ *p*-values computed from multivariable regression models fitted in the propensity score-matched sample incorporating matching variables as interaction terms; *p*-values were adjusted for multiple comparisons using the modified Bonferroni correction method described by Hochberg (1988)

§ Marginal odds ratio computed from quasi-binomial regression models (in the propensity score-matched sample) exploring the association of outcome variable with the grouping variable (administration of catheter-directed thrombolysis) incorporating matching variables as interaction terms for a doubly robust estimate

AT=Acceleration time; *FAC*=fractional area change; *IQR*=interquartile range; *mM*=marginal mean; *mOR*=marginal odds ratio; *PASP*=pulmonary artery systolic pressure; *PW*=pulse-wave Doppler; *RA*=right atrium; *RAP*=RA pressure; *RV*=right ventricle; *RVOT*=right ventricular

outflow tract; *TAPSE*=tricuspid annular plane systolic excursion; *TR*=tricuspid regurgitation; V_{max}=maximum velocity.

Propensity score-matched analysis for surgical pulmonary embolectomy (SPE)

For investigating the association between echocardiographic parameters and SPE, a propensity score-matched analysis was performed. Patients who had received SPE were designated as the target group and patients in the control group (those who had not received *any* advanced therapy) were matched on the propensity score to one or more patients in the target group using the optimal *full* matching specification—with the desired estimand being the average treatment effect ("ATE") in the population. The effective sample size of the propensity score-weighted sample was 76.7. Love plot showed improved balance overall in the matched sample (Supplementary Figure S7).

The median values of echocardiographic parameters on initial ("pre") and follow-up ("post") studies in the propensity score-matched sample are provided in Supplementary Table S3. Patients who received SPE had significant improvement in echocardiographic parameters, both on quantitative analyses (Supplementary Figure S8) and qualitative analyses (Supplementary Figure S9). Most notably, patients who underwent SPE had higher odds of normalization of TR V_{max} (marginal OR: 5.579; p<0.001) and PASP (marginal OR: 5.773; p<0.001) compared to the control group.

In terms of clinical outcomes, patients who underwent SPE had higher odds of 30-day major bleeding (marginal OR: 5.149; p<0.001) as compared to patients who did not receive any ART. However, hospital LOS (marginal mean: +3.300; p=0.090) did not differ significantly for patients who underwent SPE *vs.* those who did not undergo SPE.

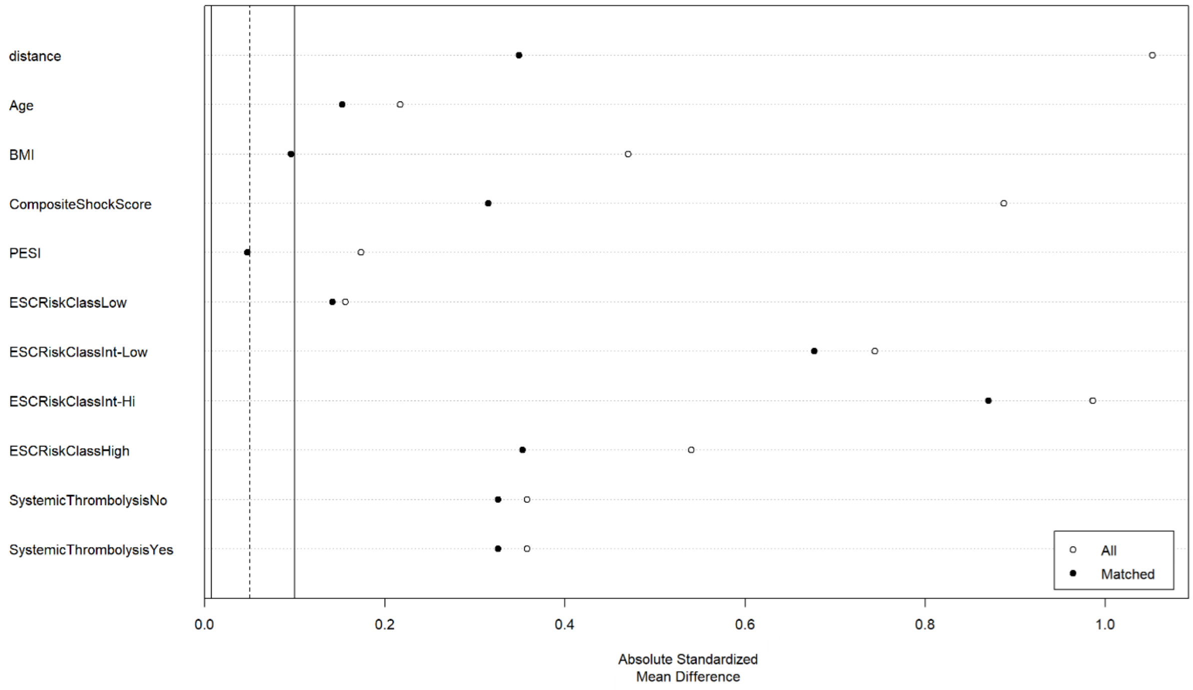

Supplementary Figure S7: Love plot depicting the absolute standardized mean differences on the matching variables (age, BMI, PESI score, ESC risk group, Composite PE Shock score and systemic thrombolysis) between the target (patients who underwent SPE) and the control (patients who did not receive any advanced therapy) groups. *BMI*=Body mass index; *CDE*=catheter-directed embolectomy; *ESC*=European Society of Cardiology; *PE*=pulmonary embolism; *PESI*=PE Severity Index.

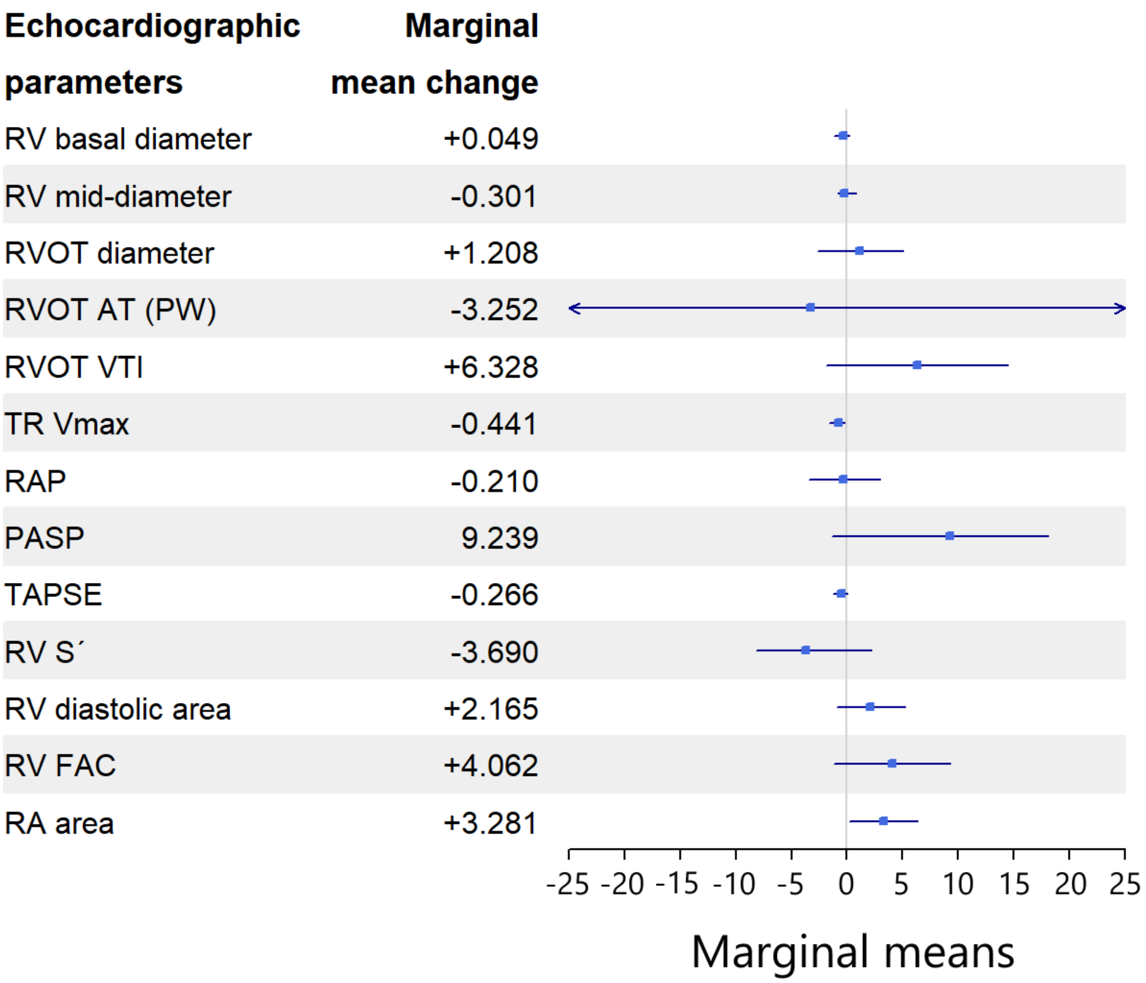

Supplementary Figure S8: Forest plot depicting the marginal means of change in echocardiographic parameters on follow-up echocardiography among patients who received SPE *vs.* those who did not receive any advanced therapy in the propensity score-matched sample. *AT*=Acceleration time; *FAC*=fractional area change; *PASP*=pulmonary artery systolic pressure; *PW*=pulsed-wave Doppler; *RA*=right atrium; *RAP*=right atrial pressure; *RV*=right ventricle; *RVOT*=right ventricular outflow tract; *S´*=lateral tricuspid annulus peak systolic velocity (on tissue Doppler); *TAPSE*=tricuspid annular plane systolic excursion; *TR*=tricuspid regurgitation; V_{max}=maximum velocity; *VTI*=velocity time integral.

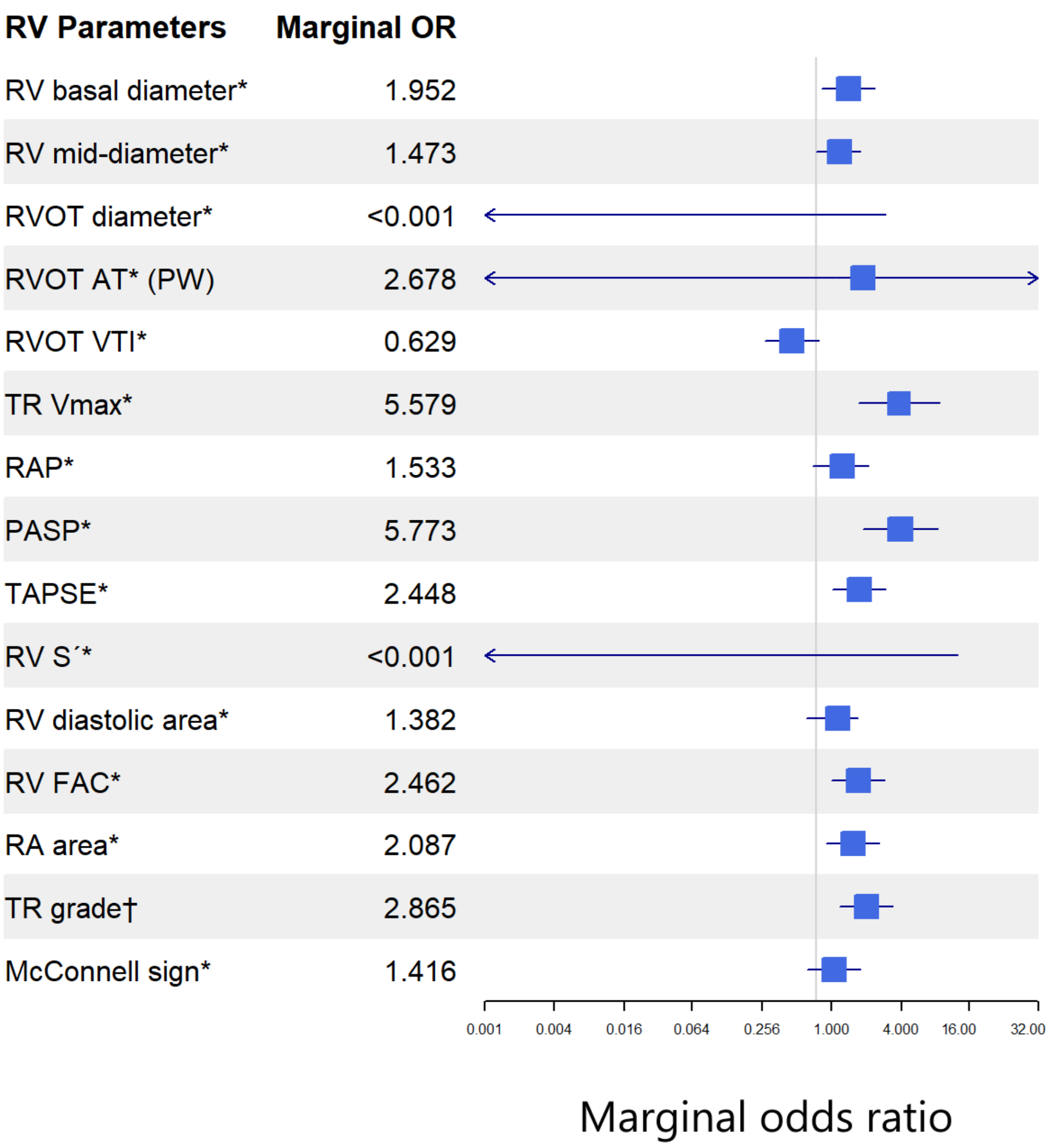

Supplementary Figure S9: Forest plot depicting the marginal odds ratios for normalization of various echocardiographic parameters on follow-up echocardiography among patients who received SPE *vs.* those who did not receive any advanced therapy in the propensity score-matched sample. *AT*=Acceleration time; *FAC*=fractional area change; *FVE*=flow velocity envelope; *OR*=odds ratio; *PASP*=pulmonary artery systolic pressure; *PW*=pulsed-wave Doppler;

RA=right atrium; *RAP*=right atrial pressure; *RV*=right ventricle; *RVOT*=right ventricular outflow tract; *S´*=lateral tricuspid annulus peak systolic velocity (on tissue Doppler); *TAPSE*=tricuspid annular plane systolic excursion; *TR*=tricuspid regurgitation; V_{max}=maximum velocity; *VTI*=velocity time integral. * Marginal odds for normalization of echocardiographic parameter; † marginal odds for improvement in TR grade.

Supplementary Table S3: Echocardiographic parameters for the intervention (surgical pulmonary embolectomy) and control groups in the propensity score-matched sample (n=122)

QUANTITATIVE PARAMETERS	GROUP	PRE	POST	mM†	*p*‡
		Median* (IQR)	Median* (IQR)		
RV basal diameter (cm)	Intervention	3.10 (3.10–4.17)	3.66 (3.62–3.70)	+0.049	0.776
	Control	4.27 (3.76–4.77)	3.64 (3.25–4.23)		
RV mid-diameter (cm)	Intervention	2.60 (2.60–3.47)	2.55 (2.51–2.59)	**-0.301**	**0.015**
	Control	3.34 (2.90–3.75)	2.80 (2.31–3.20)		
RVOT diameter (cm)	Intervention	2.30 (2.08–2.52)	2.42 (2.30–2.54)	+1.208	0.541
	Control	2.52 (2.13–2.97)	2.41 (2.15–2.80)		
RVOT PW AT (msec)	Intervention	50.6 (50.2–51.2)	96.6 (96.2–97.3)	**-3.252**	0.802
	Control	71.1 (50.7–90.0)	78.5 (56.4–108.0)		
RVOT VTI (cm)	Intervention	12.3 (11.9–12.7)	16.1 (15.9–16.3)	+6.328	0.128
	Control	9.56 (7.29–13.2)	13.5 (10.9–17.7)		
TR jet V_{max} (m/s)	Intervention	3.65 (3.12–3.68)	2.06 (2.02–2.10)	**-0.441**	**0.004**
	Control	2.91 (2.45–3.46)	2.52 (2.21–3.01)		
RAP (mm Hg)	Intervention	3.15 (2.16–4.14)	5.0 (5.0–5.0)	-0.210	0.898
	Control	5.0 (5.0–10.4)	2.96 (1.43–4.48)		
PASP (mm Hg)	Intervention	55.4 (45.0–57.6)	22.0 (21.4–22.6)	+9.239	0.401
	Control	35.0 (26.6–48.6)	35.0 (26.6–48.6)		
TAPSE (cm)	Intervention	1.64 (1.61–1.68)	1.44 (1.26–1.68)	-0.266	0.053
	Control	1.84 (1.30–2.09)	1.98 (1.74–2.31)		
RV S´ (cm/s)	Intervention	9.45 (9.18–9.73)	9.82 (9.62–10.50)	-3.690	0.112
	Control	11.9 (8.97–14.39)	11.7 (9.87–14.0)		
RV diastolic area (cm^2)	Intervention	14.4 (14.1–20.7)	14.1 (14.1–15.6)	+2.165	0.161
	Control	23.6 (19.5–29.0)	18.5 (14.7–23.8)		
RV FAC (%)	Intervention	26.0 (25.9–26.6)	36.1 (35.3–37.2)	+4.062	0.130
	Control	29.5 (21.1–36.8)	36.9 (29.4–43.9)		

RA Area (cm^2)		Intervention	13.0 (13.0–13.1)		16.3 (15.8–16.8)		+3.281	0.035
		Control	17.1 (14.1–21.2)		15.6 (11.8–17.8)			
**QUALITATIVE PARAMETERS**		**GROUP**	**PRE**		**POST**		**mOR§**	***p*‡**
			**N***	**%**	**N***	**%**		
McConnell sign		Intervention	8.6	86.0%	0	0.0%	1.445	0.182
		Control	40.1	60.1%	7.3	11.0%		
TR grade	None	Intervention	1.0	9.7%	1.0	9.7%	**2.865**	**<0.001**
		Control	9.4	14.5%	21.1	31.2%		
	Mild	Intervention	2.3	22.6%	9.0	90.3%		
		Control	36.3	56.3%	35.3	52.3%		
	Moderate	Intervention	6.8	67.7%	0.0	0.0%		
		Control	11.9	18.4%	5.6	8.3%		
	Severe	Intervention	0.0	0.0%	0.0	0.0%		
		Control	6.9	10.7%	5.6	8.3%		

* Weighted medians and frequencies computed in the propensity score-matched sample

† Marginal mean of Δ (echocardiographic parameter on follow-up echocardiography subtracted from echocardiographic parameter on baseline echocardiography) based on linear regression models (in the propensity score-matched sample) exploring the association of Δ with the grouping variable (administration of surgical pulmonary embolectomy) incorporating matching variables as interaction terms for a doubly robust estimate

‡ *p*-values computed from multivariable regression models fitted in the propensity score-matched sample incorporating matching variables as interaction terms; *p*-values were adjusted for multiple comparisons using the modified Bonferroni correction method described by Hochberg (1988)

§ Marginal odds ratio computed from quasi-binomial regression models (in the propensity score-matched sample) exploring the association of outcome variable with the grouping variable (administration of surgical pulmonary embolectomy) incorporating matching variables as interaction terms for a doubly robust estimate

AT=Acceleration time; *FAC*=fractional area change; *IQR*=interquartile range; *mM*=marginal mean; *mOR*=marginal odds ratio; *PASP*=pulmonary artery systolic pressure; *PW*=pulse-wave Doppler; *RA*=right atrium; *RAP*=RA pressure; *RV*=right ventricle; *RVOT*=right ventricular

outflow tract; *TAPSE*=tricuspid annular plane systolic excursion; *TR*=tricuspid regurgitation; V_{max}=maximum velocity.
